# Ultrastructural insight into SARS-CoV-2 entry and budding in human airway epithelium

**DOI:** 10.1038/s41467-022-29255-y

**Published:** 2022-03-25

**Authors:** Andreia L. Pinto, Ranjit K. Rai, Jonathan C. Brown, Paul Griffin, James R. Edgar, Anand Shah, Aran Singanayagam, Claire Hogg, Wendy S. Barclay, Clare E. Futter, Thomas Burgoyne

**Affiliations:** 1 Royal Brompton Hospital, Guy’s and St Thomas’ NHS Foundation Trust, London, SW3 6NP UK; 2 Department of Infectious Disease, Imperial College London, London, W2 1PG UK; 3 Department of Pathology, University of Cambridge, Cambridge, CB2 1QP UK; 4 MRC Centre of Global Infectious Disease Analysis, Department of Infectious Disease Epidemiology, School of Public Health, Imperial College London, London, W2 1PG UK; 5 Centre for Molecular Bacteriology and Infection, Imperial College London, London, SW7 2DD UK; 6 Academic Health Sciences Centre, Imperial College, London, London, SW3 6LY UK; 7 UCL Institute of Ophthalmology, University College London, London, EC1V 9EL UK

**Keywords:** SARS-CoV-2, Mechanisms of disease

## Abstract

Ultrastructural studies of SARS-CoV-2 infected cells are crucial to better understand the mechanisms of viral entry and budding within host cells. Here, we examined human airway epithelium infected with three different isolates of SARS-CoV-2 including the B.1.1.7 variant by transmission electron microscopy and tomography. For all isolates, the virus infected ciliated but not goblet epithelial cells. Key SARS-CoV-2 entry molecules, ACE2 and TMPRSS2, were found to be localised to the plasma membrane including microvilli but excluded from cilia. Consistently, extracellular virions were seen associated with microvilli and the apical plasma membrane but rarely with ciliary membranes. Profiles indicative of viral fusion where tomography showed that the viral membrane was continuous with the apical plasma membrane and the nucleocapsids diluted, compared with unfused virus, demonstrate that the plasma membrane is one site of entry where direct fusion releasing the nucleoprotein-encapsidated genome occurs. Intact intracellular virions were found within ciliated cells in compartments with a single membrane bearing S glycoprotein. Tomography showed concentration of nucleocapsids round the periphery of profiles strongly suggestive of viral budding into these compartments and this may explain how virions gain their S glycoprotein containing envelope.

## Abstract (web summary)

Human airway epithelium infected with SARS-CoV-2 shows viral entry occurring at the apical plasma membrane, excluding cilia. Here, the visualization of viral fusion at the cell surface and budding profiles within infected cells through high-resolution electron microscopy and tomography provide new insight into the infection process.

## Introduction

Severe acute respiratory syndrome coronavirus-2 (SARS-CoV-2) emerged as a novel virus in late 2019, causing widespread infections within a short period of time leading to a global pandemic. The high affinity of SARS-CoV-2 Spike (S) glycoprotein for human angiotensin-converting enzyme 2 (ACE2) receptor has been proposed as the likely cause of the rapid spread of infection^1^. ACE2 is expressed at the apical surface of primary airway epithelial cells, with expression levels showing a gradient from upper to lower airway^2^. S glycoprotein forms homotrimers emanating from the viral surface and comprises the S1 subunit with a receptor-binding domain (RBD) that binds to ACE2 and the S2 subunit that mediates fusion of viral and cellular membranes^3^.

The serine protease TMPRSS2 that is also expressed within the respiratory epithelium has been implicated in viral entry by cleaving S glycoprotein^4^. Upon binding to ACE2, the prefusion S1/S2 complex is proteolytically cleaved at the second site in S2 (S2′) by TMPRSS2, which triggers dissociation of S1 and a conformational shift in S2 to give the post-fusion form of S glycoprotein^5^. Fusion of viral membrane to the host cell plasma membrane has been shown to be driven by this cleaved membrane-anchored S2 subunit^6^. After membrane fusion occurs, the nucleocapsid protein complexed with viral RNA are released from the viral lumen into the cytoplasm of the host cell to initiate viral replication.

SARS-CoV-2 is able to enter cells lacking TMPRSS2 by alternative pathways^4,7,8^. An endocytosed virus is trafficked to endosomes that eventually fuse with lysosomes and/or are engulfed by autophagosomes^9^. Endocytic cysteine proteases cathepsins B and L can cleave the S2′ site to gain a post-fusion equivalent form of S glycoprotein^5,10^. There is evidence that two-pore channels can facilitate the release of viral RNA from endolysosomes to allow for viral replication^11^.

Virus replication is believed to take place at perinuclear sites where like for other positive-sense RNA viruses, endoplasmic reticulum (ER)-derived structures known as double-membrane vesicles (DMVs) form^12^. The DMVs are interconnected by convoluted membranes and small open double-membrane spherules (DMSs)^13^. DMVs are enriched in double-stranded RNA and are associated with viral replication^14^. SARS-CoV-2 viral budding likely occurs at ER-to-Golgi intermediate compartments (ERGIC) as detected in SARS-CoV^15^. It has also been suggested that the virus can exploit lysosomes to allow egress out of the host cell post viral replication^16^. The precise route from DMV to viral budding to egress of budded virions from the cell remains to be fully established. Cleavage at the S1/S2 junction may occur during trafficking by host furin-like enzymes before reaching the cell surface^17^. There is evidence this pre-cleavage of spike enhances entry into respiratory tissue and that variants that lack the furin cleavage site use the endosomal pathway for entry and are inefficiently transmitted by the respiratory route^8^.

Novel SARS-CoV-2 variants have emerged with genetic constellations that include mutations in S glycoprotein. This raises concern as there is potential for differences in transmissibility rates, the severity of disease, and resistance to neutralising antibodies. Lineage B.1.1.7 (Variant of Concern 202012/01) is a variant that emerged in the UK on September 2020^18^. By early 2021, B.1.1.7 had become the dominant lineage in the UK, likely due to its increased transmissibility^19^. Twenty-three mutations have been identified across the viral genome, including nine associated with S glycoprotein including N501Y in RBD, ∆69-70, ∆144 in NTD, and P681H close to the furin cleavage site^18,20^. Contemporaneous lineages which do not demonstrate the apparently increased transmissibility of B.1.1.7 include B.1.258 has Δ69-70 and N439K mutations and B.1.117.19 with an A222V mutation in S glycoprotein^20^.

In this study, we examined the ultrastructure of human respiratory epithelial cells infected with three different isolates of SARS-CoV-2 (B.1.1.7, B.1.258, and B.1.117.19). No differences in the characteristics of infection were observed but the similarities provide insight into viral attachment, entry, and budding in human airway epithelium (HAE).

## Results

### SARS-CoV-2 isolates show similar characteristics upon infection of human airway cells

**Fig. 1:**
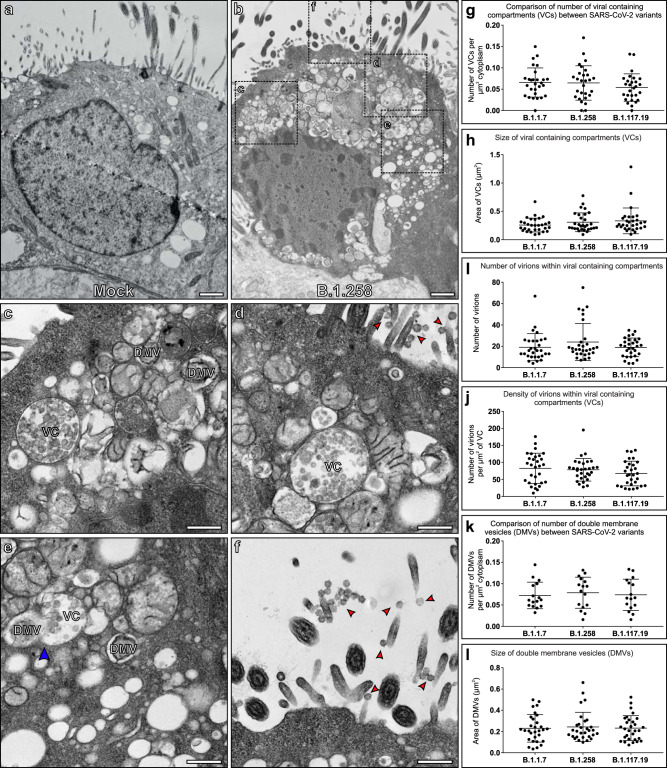
Human nasal respiratory epithelium infected with SARS-CoV-2 show considerable remodelling to give way to viral replication. **a** A non-infected ciliated epithelial cell. **b** A cell infected with B.1.258 SARS-CoV-2 variant contains a large number of viral associated compartments shown at higher magnification in (**c**–**f**) and virions at the cell surface (red arrowheads) (**d**, **f**). **c**–**e** Viral-containing compartments (VCs) and double-membrane vesicles (DMVs) can be seen. **e** Some DMVs are connected to VCs (blue arrowhead) and there are convoluted membranes surrounding these compartments. **g**–**l** Quantitation of features of VCs and DMVs from the three variants that include; **g** number of VCs within host cells, **h** size of VCs, **i** number of virions within VCs, **j** density of virions within VCs, **k** number of DMVs within host cells, and **l** size of DMVs. **g** *N* = 28 and **k** *N* = 16 cells were examined and **h**–**j**, **l** *N* = 30 VCs or DMVs were analysed. For measurements, no statistical significance was determined using paired, two-tailed Student’s *t*-tests. **g**–**l** Data were presented as mean values ± SEM. Scale bars (**a**, **b**) 1 μm and (**c**–**f**) 400 nm.

The nose is the primary site of SARS-CoV-2 infection and therefore nasal primary human airway epithelial (HAE) cells are the optimal cell culture model to simulate SARS-CoV-2 infection of the airway. Differentiation of cells at an air-liquid interface (ALI) results in an epithelial layer composed of ciliated, goblet, and basal cells. We previously infected HAE cells with a panel of SARS-CoV-2 isolates at an multiplicity of infection (MOI) of 0.01 pfu/cell and demonstrated robust viral replication kinetics in these cells with abundant infectious virus released at the apical surface at 72 h postinfection^20^. Fixation of these samples at 72 h postinfection and observation by transmission electron microscopy (TEM) showed the presence of SARS-CoV-2 virions and major changes in cellular morphology as shown in Fig. 1. As described in previous studies, host cell membrane remodelling to give way to the viral replication machinery was observed and included DMVs, convoluted membranes (CMs), and viral-containing compartments (VCs) (Fig. 1c, d)^12,21^. Some DMVs were seen to be connected to VCs as shown by the blue arrowhead in Fig. 1e. At the 72 h postinfection time point, substantial numbers of virions visible in the extracellular space were attached to the plasma membrane (red arrowheads Fig. 1d, f) consistent with the high viral titre observed in washings of the cells at this time^20^. Given the initial low MOI with which the cells were treated this must have been virus resulting from replication and release from infected cells potentially attaching to initiate further rounds of infection. Quantitation of VCs and DMVs within infected ciliated cells, including the number, size and location of these organelles as well as the number and density of virions within VCs showed no significant difference between the variants (Fig. 1g–l and Supplementary Fig. 1).

**Fig. 2:**
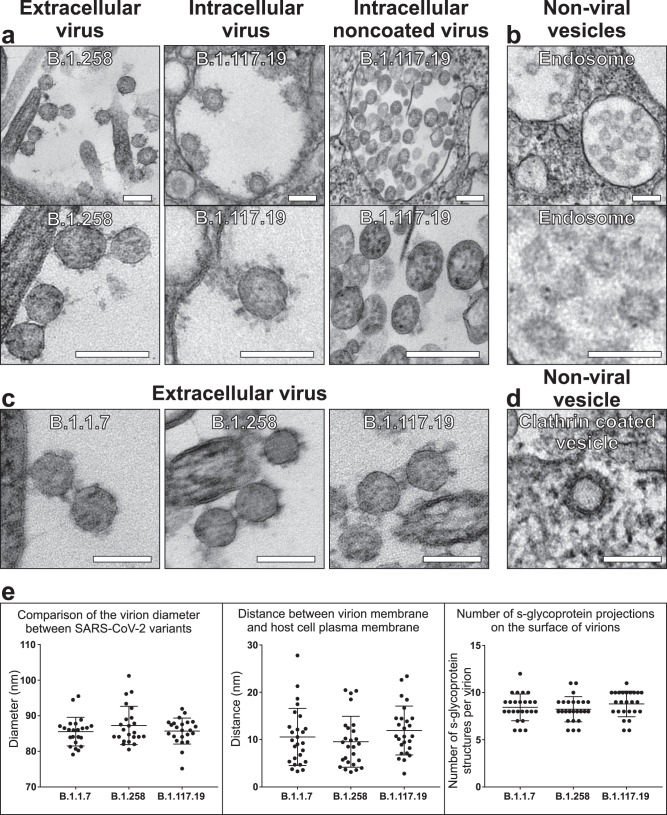
By TEM, SARS-CoV-2 virions are discernible by the punctate pattern of the nucleocapsid containing lumen and often the presence of a S glycoprotein coat. **a** Virions are seen extracellularly at the cell surface and intracellularly within viral-containing compartments. In some compartments, virions were observed without an S glycoprotein coat. **b** In comparison, endosomes consist of intraluminal vesicles that are smaller, lighter in appearance, and lack the nucleocapsid punctate interior of virions. **c** The three different lineages (B.1.1.7, B.1.258, and B.1.117.19) have a similar ultrastructural appearance by TEM. **d** A clathrin-coated vesicle can be discerned from a virion by having a denser coat that viral S glycoprotein and lacking punctate nucleocapsid in the interior. **e** There was no statistical significance in the diameter of virions (*N* = 23), the distance of virions from the host plasma membrane (*N* = 26), or the number of S glycoprotein projections on the viral surface (*N* = 25). This was determined using paired, two-tailed Student’s *t*-tests. Data were presented as mean values ± SEM. Scale bars (**a**–**d**) 100 nm.

One of the identifiable traits of SARS-CoV-2 virions by TEM is the nucleocapsid contents of the viral lumen that present as an electron-dense punctate pattern, making them distinguishable from clathrin-coated and intraluminal vesicles (Fig. 2). The SARS-CoV-2 spike (S) glycoprotein was evident on most extracellular virions as well as those in VCs (Fig. 2a). Some of the VCs contained virions that were not coated with S glycoprotein on the surface. When comparing the three isolates B.1.1.7, B.1.258, B.1.117.19, at the resolution of conventional TEM there were no detectable differences in structure, size, or attachment to the host cell (Fig. 2c, e).

### Extracellular virions mostly localise to microvilli but not cilia

**Fig. 3:**
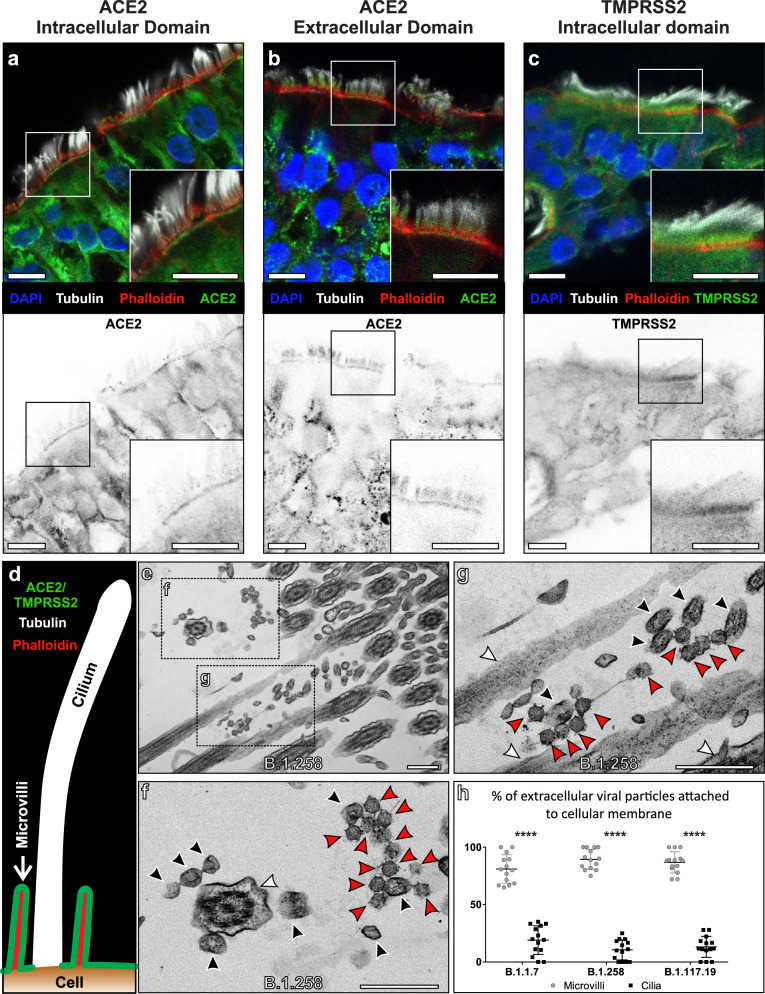
ACE2 and TMPRSS2 localises to the plasma membrane excluding cilia and this coincides with the sites of viral attachment. **a**–**c** Immunofluorescence antibody labelling against ACE2 and TMPRSS2 using anti-tubulin as a marker for cilia and phalloidin for the actin enriched microvilli. Both ACE2 and TMPRSS2 were found to be localised to cell surface that included the microvilli. Although there is diffuse, likely nonspecific, staining everywhere with the TMPRSS2 antibody, it is clearly enriched on the apical plasma membrane. Grey scale images of the staining profiles of ACE2 and TMPRSS2 show that they only extend a short distance from the cell surface unlike the tubulin staining of cilia and overlap with the phalloidin staining. **d** A diagram of the staining pattern of the antibodies. **e**–**g** When looking by TEM, virions (red arrowheads) are seen attached to microvilli (black arrowheads) and not to cilia (white arrowheads). **h** Quantification of the number of viral particles attached (within 3 nm) to microvilli and cilia, there is a little affinity of the virus to cilia for all three isolates. Single samples were examined for each and the number of cells assessed *N* = 14. Mean and SEM shown and statistical significance was determine using paired, two-tailed Student’s *t*-tests (B.1.1.7 *P* = 5.3 × 10^−13^, B.1.258 *P* = 3.44 × 10^−19^, and B.1.117.19 *P* = 7.19 × 10^−18^). Scale bars a–c 10 μm and e–g 400 nm.

Sections of HAE were stained for the extracellular and intracellular domains of ACE2 and the intracellular domain of TMPRSS2 by immunofluorescence. This was done in combination with antibody staining against tubulin to identify cilia and phalloidin to highlight the actin enriched microvilli at the cell surface. The staining shows ACE2 and TMPRSS2 localised to regions of the plasma membrane that included microvilli but was largely excluded from cilia (Fig. 3a–d). All three antibodies stained other regions of the epithelial cells and gave some diffuse cytoplasmic staining likely to be a background. Antibody detecting the intracellular domain of ACE2 stained intensely at the base of microvilli, whereas staining with antibodies against the extracellular ACE2 domain also extended through the microvilli level. Neither antibody stained the tips of cilia. The difference in staining pattern between the two ACE2 antibodies could result from the extracellular domain antibody only detecting the larger ACE2 isoform, whereas the intracellular domain antibody is detecting the short ACE2 isoform^22^. The shorter isoform may not be localised throughout the microvilli, therefore, detection of this and the longer isoform of ACE2 at the microvilli base by the intracellular domain antibody would generate a stronger signal that is more readily detectable compared to the longer ACE2 isoform through the microvilli. A similar staining pattern was seen in a human nasal brushing biopsy showing that the protein localisation is not an artefact of cell culture (Supplementary Fig. 2). To validate the ACE2 and TMPRSS2 antibodies that were used, they were tested in HeLa cells expressing ACE2, Myc-ACE2, or HA-TMPRSS2 (Supplementary Fig. 3) and showed much stronger staining in cells expressing the corresponding antibody target. When looking at non-detergent treated sections of HAE cells there was diffuse tubulin staining due to poor accessibility of the antibody into cilia, but the antibody raised against the extracellular domain of ACE2 still provided a strong signal. This was particularly evident in a confocal slice of apical epithelium where cilia were absent and ACE2 staining was enriched in regions densely populated with phalloidin stained microvilli (Supplementary Fig. 4a). When using an antibody against Ezrin, an actin-binding component of the microvilli, the staining surrounded the actin (phalloidin stained) in a pattern very similar to the antibody against the extracellular domain of ACE2 (Fig. 3b and Supplementary Fig. 4). Consistent with the localisation of key entry molecules on the plasma membrane at the base of and surrounding the microvilli but not on the cilia, TEM showed extracellular virions at the surface of ciliated epithelial cells mostly in contact with microvilli but not cilia (Fig. 3e–g). The preferential attachment to microvilli over cilia was observed for all three isolates (Fig. 3h). Furthermore, using an antibody against S glycoprotein, staining was found to be close to the cell surface in infected HAE cells and did not appear to overlap with cilia (Tubulin staining) (Supplementary Fig. 5).

### Viral infection is rarely detected in goblet cells

**Fig. 4:**
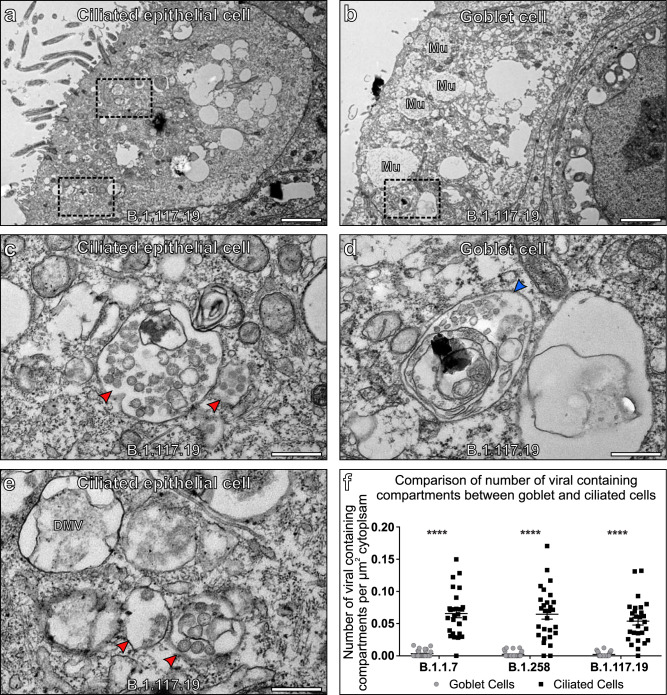
Goblet cells show little evidence of SARS-CoV-2 infection. **a**–**c** Ciliated cells contain readily identifiable viral-containing compartments (red arrowhead) and double-membrane vesicles (DMV). **d**, **e** Goblet cells contain endosomes and endolysosomes (blue arrowhead) that have a similar appearance to viral-containing compartments but can be differentiated by the lack of viral nucleocapsid protein and S glycoprotein structures. Goblet cells also contain mucin-containing secretory granules (Mu). **f** Very few VCs were found in goblet cells for all three isolates. Single sample for each examined and the number of cells assessed *N* = 28. Mean and SEM are shown, and statistical significance was determine using paired, two-tailed Student’s *t*-tests (B.1.1.7 *P* = 3.48 × 10^−13^, B.1.258 *P* = 6.07 × 10^−11^, and B.1.117.19 *P* = 1.81 × 10^−11^). Scale bars (**a**, **d**) 2 μm and (**b**, **c**, **e**) 400 nm.

VCs can be identified by TEM by the distinct morphology of virions contained within (Figs. 1 and Fig 4a–c). VCs can be used as a marker of cellular infection as these will have resulted from successful viral entry and genome replication within the host cell. Few VC or VC-like compartments were identified when examining goblet cells that are characterised by the presence of mucin-containing secretory granules (Mu in Fig. 4d). Endosome and endolysosomes (Fig. 4e) were observed in this cell type and could be distinguished from VCs by the intraluminal vesicles having a smaller diameter, less electron-dense contents, and lacking S glycoprotein (Fig. 2). The lack of VCs in goblet cells in comparison to ciliated cells was consistent for all isolates (Fig. 4f) and likely due to lack of viral infection because the majority of goblet cells had neither virus particles at the cell surface nor identifiable DMVs (Supplementary Fig. 6). This is in contrast to ciliated epithelial cells all of which contained DMVs (see Fig. 1) and even in the very few cells where surface virus or DMVs could be detected the numbers/cells were far lower than in ciliated epithelia.

### S glycoprotein-like structures on the plasma membrane of ciliated cells are a marker of infected cells

**Fig. 5:**
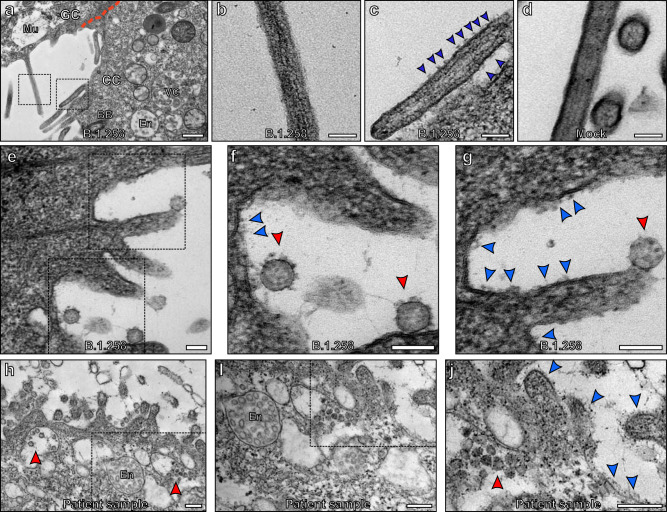
S glycoprotein-like protrusions were found on microvilli and plasma membrane of infected cells. **a** An uninfected goblet cell (GC) next to an infected ciliated cell (CC) that has viral-containing compartments (VC) (endosome (En) and basal bodies (BB) were also visible). The goblet cell is identifiable by the mucin-containing secretory granules (Mu) and the junction between the two cells false coloured with a red line. When looking at the microvilli from the goblet cell shown in (**a**) and at higher magnification (**b**), the membrane is smooth. Conversely, the plasma membrane of the microvilli of the infected ciliated cell (**a**) and at higher magnification (**c**), is not smooth and has protrusions (blue arrowheads). **d** The microvilli of a non-infected ciliated cell are smooth. **e** High magnification images of the boxed regions (**f**, **g**) show infected cultured human airway epithelial cells where viral particles can be seen attached to the cell surface. The viral S glycoprotein (red arrowheads) has a similar profile to the cell surface protrusions (blue arrowheads). **h**–**j** Cells acquired from a nasal brushing of an infected patient show the presence of viral particles (red arrowheads). **I**, **j** At higher magnification protrusions are seen at the cell surface and on microvilli (blue arrowheads). Scale bars (**a**) 400 nm, (**b**–**g**) 100 nm, and (**h**–**j**) 200 nm.

Another marker of infected cells was the presence of S glycoprotein protein-like structures at the plasma membrane. These were found on microvilli of infected ciliated cells but were absent from the microvilli of uninfected goblet cells (Fig. 5). This feature can be seen when comparing a ciliated cell neighbouring a goblet cell (Fig. 5a–c). The goblet cell has microvilli with a smooth plasma membrane similar to that of an uninfected ciliated cell (Fig. 5b, d). S glycoprotein-like protrusions are clearly visible on the infected ciliated cells microvilli (blue arrows in Fig. 5c and Supplementary Fig. 7a). Protrusions were absent from the cilia of infected cells as shown in Supplementary Fig. 7b. Areas of the plasma membrane that have attached virions, also have protrusions with similar morphology to the viral S glycoprotein (blue and red arrows in Fig. 5e–g). This was consistent with antibody labelling against S glycoprotein by immune electron microscopy (iEM) that localised to microvilli and the cell surface of infected cells but not to cilia (Supplementary Fig. 5). S glycoprotein at the cell surface could be a result of virus fusing with the plasma membrane and leaving behind S glycoprotein in the fused membrane patch or S glycoprotein that has been expressed in an infected cell and transported to the plasma membrane. When examining cells from a nasal brushing of a SARS-CoV-2 infected patient (unknown viral lineage), similar protrusions on the plasma membrane and microvilli could be seen (Fig. 5h–j). In non-infected patients, these protrusions were absent as seen in Supplementary Fig. 7c.

### Viral fusion at the plasma membrane

**Fig. 6:**
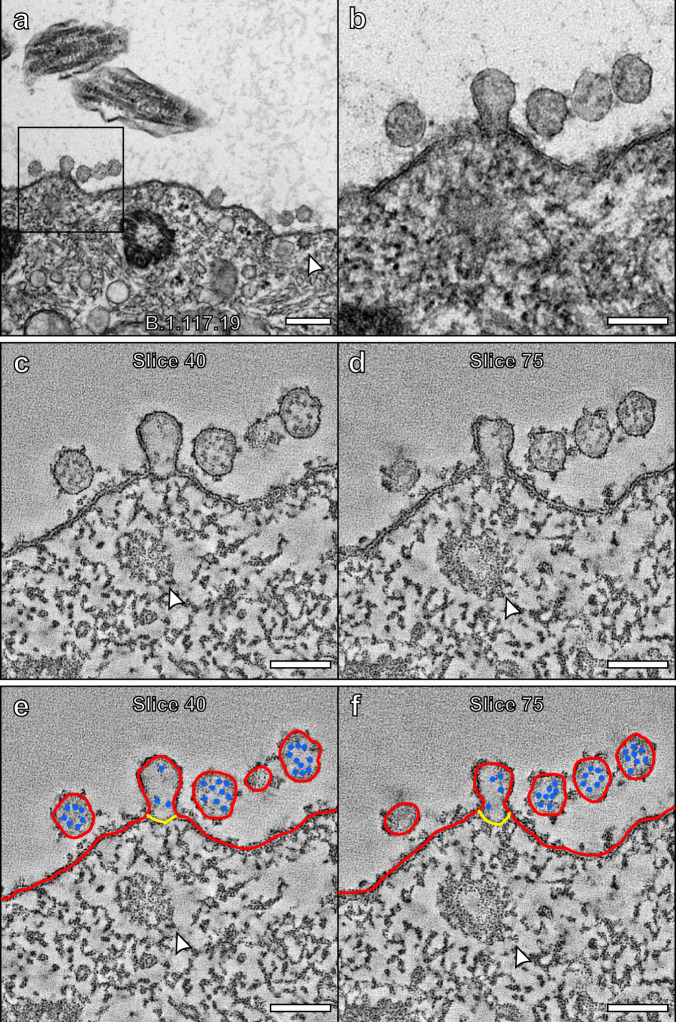
SARS-CoV-2 virion fused to the plasma membrane and released its contents into a ciliated cell. **a** TEM image of a virion fused to a host cell and higher magnification of the boxed region (**b**). **c**–**f** Tomogram generated of the attached virion with false colours (**e**, **f**) to indicate viral and plasma membrane (red), nucleocapsid protein (blue), and a neck-like structure (yellow). **e**, **f** The viral membrane is continuous with the host cell plasma membrane and the nucleocapsid contents are diluted as they are released into the host cell. The white arrowheads highlight **a**, **b** a clathrin-coated pit and **e**, **f** a clathrin-coated vesicle. Scale bars **a** 200 nm and **b**–**f** 100 nm.

TEM coupled with electron tomography allowed the capture of profiles strongly indicative of viruses fusing at the host ciliated cell plasma membrane (Fig. 6 and Supplementary Movie 1, with further examples shown in Supplementary Fig. 8). Figure 6a, b show virions close to the apical plasma membrane and one virus that appears to have fused with the host cell plasma membrane. To examine the fusion in greater detail a tomogram was generated that shows the membrane of the virus is continuous with the plasma membrane of the host cell (Fig. 6c, d and shown in red in Fig. 6e, f). The nucleocapsid protein content within this virion is diluted compared to that of whole virions in the vicinity (false coloured in blue in Fig. 6e, f), indicating that some have already entered the host cell, consistent with this profile indicating viral fusion rather than budding. This was supported by iEM labelling of nucleocapsid protein that localised within virions and at the surface of infected cells that also included microvilli (Supplementary Fig. 9). A neck-like structure was observed at the site of fusion (false coloured in yellow in Fig. 6e, f).

### Viral budding within infected cells

**Fig. 7:**
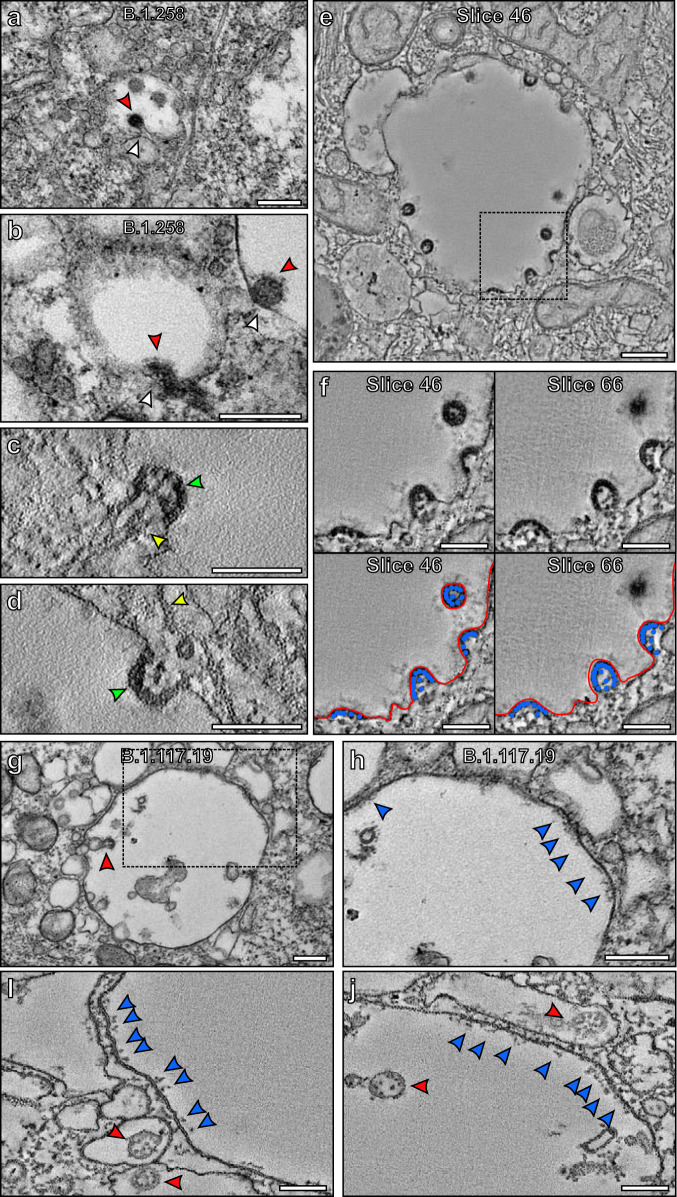
Virions appear to bud into VCs coated in S glycoprotein that are transferred from the limiting membrane. **a**, **b** Virions (red arrowheads) appear to be budding into a VC which has a S glycoprotein coat. **c**–**f** Slices from tomograms indicating budding at the limiting membrane of a VC. Microtubules were seen running into budding virions (**c**) or nearby (**d**) as indicated by the yellow arrowheads. The boxed region in (**e**) is shown at higher magnification in (**f**) that includes two different slices from a tomogram. The nucleocapsid protein appeared to be concentrated at the membrane of the budding virions (false coloured blue in (**f**) and green arrowhead in (**c**, **d**)). The boxed region in (**g**) is shown at higher magnification (**h**) of a VC that contains a virus (red arrowhead) and has S glycoprotein (blue arrowheads) on the limiting membrane facing the lumen. **i**, **j** Slices from tomograms generated of VCs showing S glycoprotein on the limiting membrane in more detail. Viral particles were within the VCs as well as surrounded by membranes in structures close by. Scale bars (**a**, **b**, **e**, **g**, **h**) 200 nm and (**c**, **d**, **f**, **i**, **j**) 100 nm.

Within VCs, virions were observed to be likely budding from the limiting membrane (Fig. 7a, b). Some had neck-like structures that resembled a stalk (unlike the neck structure found for fusing virions at the site of viral attachment to the plasma membrane), indicating that the virion is pulling away in the direction of the VC lumen as shown in Fig. 7a. This implies that it is likely to be budding from rather than fusing with the membrane, even though fusion cannot be ruled out. As these virions emerge from the membrane, they are clearly coated with S glycoprotein (Fig. 7a, b). A tomogram of a VC containing two budding virions (Supplementary Movie 2) shows a network of microtubules running into or near the emerging virions (yellow arrowhead in Fig. 7c, d). These could have a role in transporting viral components such as nucleocapsid protein and RNA to the site of viral budding. The electron-dense nucleocapsid protein appeared to be concentrated at the periphery of the budding virions against the viral membrane, potentially driving the budding process (green arrowhead in Fig. 7c, d, false coloured in blue in Fig. 7e, f and Supplementary Fig. 10 with Movies of tomograms shown in Supplementary Movies 2–4). The packing of the nucleocapsid protein appears more diffuse in discrete virions compared to those that have a budding profile (Fig. 7e, f and Supplementary Fig. 10). This appearance of the nucleocapsid protein is different from that of the virion fused at the plasma membrane where it was found to be diluted (Fig. 6), providing further evidence that these are budding profiles within VCs. Within VCs, S glycoprotein-like protrusions facing the lumen were observed, similar to the structures found on the plasma membrane of infected cells (Fig. 7g, h). To examine these structures further, tomograms were generated (Fig. 7i, j and Supplementary Movies 5–7). These showed the structure of these protrusions in greater detail and along with iEM labelling (Supplementary Fig. 5), confirmed they are likely to be S glycoprotein. Virions were also visualised within irregular-shaped membrane structures in close contact with the VCs (red arrowheads in Fig. 7) that appear to be convoluted membranes^12^.

### Structural comparison of S glycoprotein and membrane protrusions

**Fig. 8:**
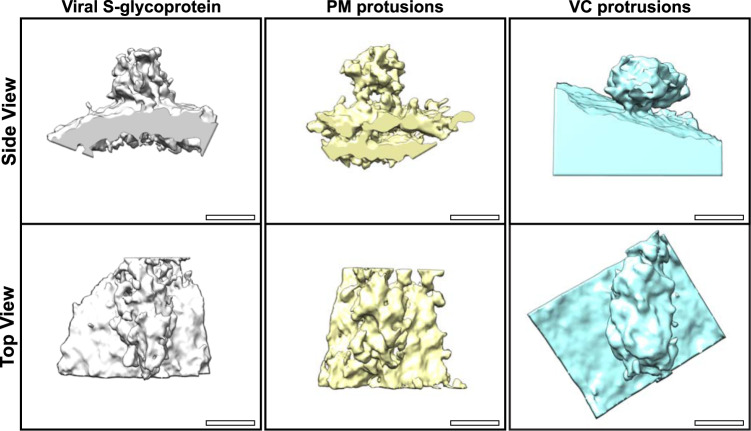
Subtomographic averages of the plasma membrane (PM) and viral compartment (VC) protrusion have a similar structure to viral S glycoprotein. Scale bar 10 nm.

To further assess the structures of the protrusions at the plasma membrane and within VCs and to compare to viral S glycoprotein, subtomographic averages were generated. This approach removes the background and enhances the 3D resolution of the tomography data as shown in Fig. 8. All three reconstructions had a strong resemblance that included a globular-like structure protruding from either the viral, plasma or VC membrane. We also transfected HeLa cells with ss-HA-S glycoprotein and again observed protrusions at the plasma membrane that correlated with S glycoprotein iEM labelling (Supplementary Fig. 11). Based on the 3D structures and presence of protrusions in ss-HA-S glycoprotein expressing HeLa cells it is highly likely that these are all S glycoprotein.

## Discussion

Electron microscopy techniques allow high magnification and detailed visualisation of viral processes that cannot be obtained using other methods. By examining primary human airway epithelial (HAE) cells infected with three different isolates of SARS-CoV-2 we are able to show aspects of viral attachment, entry, and likely budding that have not been previously described. Due to the severity and global impact of SARS-CoV-2, it is essential to understand these processes as the mechanisms underlying them present potential therapeutic targets.

For both SARS-CoV and SARS-CoV-2, ACE2 is a key host molecule expressed in airway cells required for viral entry through interaction with S glycoprotein^4,23,24^. In addition to engagement with the ACE2 receptor, SARS-CoV-2 utilises a second host molecule, TMPRSS2, that cleaves the S glycoprotein S2 subunit to allow membrane fusion and release of viral contents into the host cell. Previous studies have indicated ACE2 is localised to the cilia plasma membrane, which implies that cilia would be the likely site of extracellular virion attachment^25^. In contrast, we show here both ACE2 and TMPRSS2 are localised to discrete areas of the ciliated cell plasma membrane that include microvilli but exclude cilia themselves. A number of factors could underlie this discrepancy, including the degree of differentiation of the cells and the sample preparation, given that the previous study used paraffin-embedded samples that require antigen retrieval unlike the cryostat sections examined here. Our conclusion that ACE2 is largely excluded from the cilia is strengthened by the ease of distinction between cilia and microvilli in our highly differentiated cells, our demonstration that these antibodies faithfully localise transfected ACE2 in cells that do not normally express it, and that the virions attach preferentially to microvilli and other regions of the plasma membrane and rarely associate with cilia.

Goblet epithelial cells that neighbour ciliated epithelial cells have microvilli but lack cilia. When comparing these cell types in infected HAEs, evidence of intracellular virus replication such as VCs and DMVs could be clearly seen in ciliated cells but were rarely found in goblet cells. Similar observations were made in a previous study looking at a seasonal coronavirus^26,27^ and for SARS-CoV-2 there is evidence for preferential targeting of ciliated cells^28^. It is important to note that only a few DMVs were observed within goblet cells, and virions were seen at the cell surface of only a small number of cells. This indicates that goblet cells could lack enough machinery for viral entry either via an extracellular route or laterally from neighbouring ciliated cells at least at the early stages of infection. Furthermore, the mucus produced by these cells could be protective and prevent much of the virus from accessing the goblet cell surface. There is evidence that asthma sufferers do not have increased susceptibility to SARS-COV-2 infection and that the mucus hypersecretion in some patients could be a protective factor against COVID-19^29^. Over the 72 h of SARS-CoV-2 infection, there was extensive viral replication as indicated by a large number of extracellular virions exclusively at the surface of ciliated cells and measurement of replication kinetics in a parallel study^20^. In some studies, infection of goblet cells by SARS-CoV-2 has been documented^21,30,31^. Therefore, it is possible with an extended period of infection that the goblet cells may become vulnerable to infection.

Viral fusion at the plasma membrane is proposed as a route of viral entry for SARS-CoV-2 in airway cells^8^. We were able to capture and examine this process using the 3D technique, electron tomography. The viral membrane could be seen to be continuous with the host cell plasma membrane and its contents diluted as they likely flowed from the viral lumen into the host cell cytoplasm. A neck-like structure was seen at the site of membrane fusion that may indicate that the virus had only just fused or there is something regulating the shape of the membranes at the fusion site. After virus fusion and release of their contents, these structures must flatten otherwise a large number of fused viral structures would be seen on the plasma membrane of infected cells. As this occurs S glycoprotein that was integrated into the viral membrane would remain at the plasma membrane. We were able to visualise the presence of S glycoprotein-like structures on the surface of infected cells that could have resulted from previous viral fusion processes. Labelling of S glycoprotein (by iEM and immunofluorescence) showed that it localised to the plasma membrane of infected cells excluding cilia. This fits with much of the plasma membrane S glycoprotein resulting from viral fusion rather than synthesis and integration in the plasma membrane. Free S glycoprotein synthesised by the host would likely be localised to all regions of the plasma membrane including the cilia. S glycoprotein on the cell surface could also be derived from the fusion of VCs with the plasma membrane.

Profiles likely to be viral budding were visualised emerging from the membrane of VCs. Similar budding structures have previously been described in cell lines including Vero-81 cells, but have not been studied in primary HAEs^32,33^. Due to the profile of budding events where the bud appeared to be pulling away from the membrane in a stalk-like appearance and the position of nucleocapsid protein at the viral periphery away from the membrane opening, it is unlikely that these are fusion events. Viral budding needs to be a carefully regulated process to correctly form a virion with the necessary contents, whereas fusion of viral and host cell membranes to release viral contents into the cytoplasm does not need such regulation and will therefore likely be a much more rapid process. This is consistent with budding-like profiles readily seen within VCs that are far more frequent than the fusion profiles found at the cell surface. Even so, we cannot rule out viral fusion occurring at the VC membrane and further work is required to investigate this. The VCs appear spherical in shape when examining EM sections and some were seen to a position close to recognisable Golgi-like membrane but is difficult to be certain if they are ERGIC derived structures based on their morphology alone. Further work such as immunoelectron microscopy could help to determine the proteins localised to the VCs that have budding virions. In close proximity to the VCs, virions were found in less regular shaped membrane structures that are likely to be convoluted membranes. More work is required to determine the origin of the VCs and how virions are able to form in these proximal membranous structures. At the membrane of VCs S glycoprotein-like structures were seen facing the lumen. As virions form and bud this would allow them to gain their envelope containing S glycoprotein. If the processes that lead to expression and/or transport of S glycoprotein are not always optimal this could explain why some virions were found within VCs without an S glycoprotein coat. It is also possible that the VCs gain S glycoprotein from virions fusing at the membrane of the organelle and releasing their contents into the cell cytoplasm. Our evidence points towards budding occurring in the VCs but as we cannot completely rule out fusion or a combination of fusion and budding, future work is required to determine how VCs gain the S glycoprotein. If virions are released from host cells via fusion of VCs with the plasma membrane, any remaining S glycoprotein on the membrane would likely be transferred to the cell plasma membrane and would face the extracellular space. This could also be the source of the S glycoprotein detected at the surface of infected cells and could result in the cell-cell fusion that has been reported elsewhere by us and others^34^. Evidence was recently presented that SARS CoV-2 virions leave the cell via lysosome exocytosis^16^. Although some of the VCs have membranous inclusions, most do not have content typically associated with lysosomes. Further work is needed to determine the nature of these compartments and their relationship with both ERGIC and the lysosome.

The presence of S glycoprotein on the plasma membrane and within the lumen of cellular compartments provides an excellent marker of infected cells by electron microscopy. Based on the subtomographic averages and the presence of protrusions visible by TEM localised to the plasma membrane and within VCs after infection, in addition to iEM labelling it is highly likely that they are S glycoprotein. More work is required to determine if cell surface S glycoprotein is a result of either viral fusion with incoming particles and/or release from VCs. No difference between the isolates was detected, as higher resolution methods such as cryo-electron microscopy would be required to identify molecular differences between the S glycoproteins that only differ by nine or so amino acids. Even so, the similarities of the isolates provided an excellent overview of SARS-CoV-2 infection. There is still much more to be learned from ultrastructural analysis including the route to viral release and alternative routes of viral entry.

This study has allowed better characterisation of key processes in the life cycle of SARS-CoV-2. Cilia were found not to be the major site of key entry molecules ACE2 and TMPRSS2 and hence not involved in viral attachment and entry. In contrast, the microvilli on ciliated cells expressed abundant receptors and were a site for virion attachment followed by cell surface fusion. Goblet cells were refractory to infection. This knowledge as well as what can be gained in further studies to establish factors regulating viral budding has the potential to help in the design of novel therapeutic interventions.

## Methods

### Human nasal brushing biopsies

This study complies with ethical regulations for acquiring and use of human tissue under Health Research Authority (HRA) study approval (REC ref: 20/SC/0208; IRAS: 282739) and informed consent was obtained from the participants. Nasal brushings samples were taken from a healthy 25-year-old female, and a SARS-CoV-2 infected 61-year-old male participant turbinates using 3-mm bronchial cytology brushes. SARS-CoV-2 infection was PCR-confirmed from swabs taken at the Royal Brompton and Harefield Hospital. The cells acquired from the healthy participants’ nasal brushing were used for all in vitro infection experiments. The infected nasal brushing sample was embedded for electron microscopy and used in a single experiment as shown in Fig. 5.

### Human airway epithelium (HAE) cell culture

Nasal brushings were placed in PneumaCult-Ex Plus medium (STEMCELL Technologies, Cambridge, UK) and cells dissociated from the brush by gentle agitation. The cells were seeded into a single well of collagen (PureCol from Sigma-Aldrich) coated plate and once confluent, the cells were passaged and expanded further in a T25 flask. The cells were passaged a second time and seeded onto transwell inserts (6.5 mm diameter, 0.4 μm pore size, Corning) at a density of 24,000 cells per insert. Cells were cultured in PneumaCult-Ex Plus medium (STEMCELL Technologies, Cambridge, UK) until confluent, at which point the media was replaced with PneumaCult-ALI medium in the basal chamber and the apical surface exposed to provide an air-liquid interface (ALI). Ciliation was observed between 4–6 weeks post transition to ALI.

### HeLa cell culture and transfections

HeLa cells were seeded onto glass coverslips and transfected with either myc-ACE2 (pCEP4-myc-ACE2 from Addgene Plasmid #141185), ACE2^35^, HA-TMPRSS2 (pCSDest-HA-TMPRSS2 from Addgene Plasmid #154963), ss-HA-Spike plasmids (HA tag at the N terminus with a Serine-Glycine linker between residues S13 and Q14 of S glycoprotein^36^) using Lipofectamine 3000 (Thermo Fisher Scientific), following the manufacturer’s guidelines. Cells were fixed for immunofluorescence or electron microscopy 2 days after transfection.

### Viruses and infection of HAE cells

SARS-CoV-2 B.1.1.7 isolate hCoV-19/England/204661721/2020 (EPI_ISL_693400), B.1.258 isolate hCoV-19/England/204501206/2020 (EPI_ISL_660791), and B.1.117.19 isolate hCoV-19/England/204501194/2020 (EPI_ISL_660788) were isolated from swabs as described in ref. ^20^. Swabs were collected by the PHE Virology Consortium and ATACCC under the Integrated Network for Surveillance, Trials and Investigation of COVID-19 Transmission (INSTINCT; Ethics Ref: 20/NW/0231; IRAS Project ID: 282820). The investigation protocol was reviewed and approved by the PHE Research Ethics and Governance Group and Incident Management team. PHE has legal permission, provided by Regulation 3 of the Health Service (Control of Patient Information) Regulation 2002, to process patient confidential information for national surveillance of communicable diseases. Isolates were passaged twice in Vero cells before being used to infect HAE cells. To remove the mucus layer from the apical surface of HAE cells prior to infection, 200 ul of DMEM was added and incubated at 37 °C, 5% CO_2_ for 10 min before removal of the medium. HAE cells were then infected at a MOI of 0.01 pfu/cell of each isolate diluted in DMEM. Inocula were added to the apical chamber and incubated for 1 h at 37 °C, 5% CO_2_ before removal of the inoculum and incubating for a further 72 h. Subsequently, cells were fixed for electron microscopy.

### Conventional transmission electron microscopy (TEM)

Cultured HAE cells, nasal brushing samples, and HeLa cells were fixed by placing them in 2.5% glutaraldehyde in 0.05 M sodium cacodylate buffer at pH 7.4 and left for at least 24 h. Subsequently, the samples were incubated in 1% aqueous osmium tetroxide for 1 h at room temperature (RT) before en bloc staining in undiluted UA-Zero (Agar Scientific) for 30 min at RT. The samples were dehydrated using an increasing ethanol series (50, 70, 90, 100%), followed by propylene oxide and a mixture of propylene oxide and araldite resin (1:1). The samples were embedded by placing them in araldite and left at 60 °C for 48 h. Ultrathin sections were cut using a Reichert Ultracut E ultramicrotome and stained using Reynold’s lead citrate for 10 min at RT. Images were acquired on a JEOL 1400Plus TEM fitted with an Advanced Microscopy Technologies (AMT) XR16 charge-coupled device (CCD) camera or a Gatan Orius SC1000B CCD camera. Image acquisition software AMT Capture Engine and Gatan Digital Micrograph were used.

### Electron tomography (ET)

Electron microscopy sections were tilted and imaged within a JEOL 1400Plus TEM from ±60° over two perpendicular axes and images collect using SerialEM software (developed at University of Colorado, Boulder). Dual-axis tomograms were generated from the tilt series using IMOD (developed at the University of Colorado, Boulder)^37^. Subtomographic averaging was performed using the IMOD package, PEET (Particle Estimation for Electron Tomography). Slices from the tomograms were viewed in IMOD and the averaged structures surface rendered in Chimera (UCSF).

### Immunofluorescence (IF)

HeLa and HAE cells as well as nasal brushing biopsies were fixed in 4% PFA in PBS for 1 h. HeLa cells were stained directly whereas HAE and nasal brushings were embedded and frozen in OCT (optimal cutting temperature) compound before cutting ~15 µm thick sections using a cryostat. HeLa cells and sections were permeabilised using 0.2% saponin in PBS for 20 min at RT before incubating in blocking solution containing 0.02% saponin, 1% BSA in PBS for 30 min at RT. HeLa cells on coverslips were incubated with primary antibodies in blocking solution for 1 h at RT whereas cryostat sections were incubated overnight at 4 °C. Antibodies against α Tubulin (1:250, sc-32293, Santa Cruz and 1:250, ab52866, Abcam), ACE2 (1:100, ab15348, Abcam and 1:100, HPA000288, Sigma-Aldrich), TMPRSS2 (1:100, NBP2-38263, Novus Biologicals), HA (1:100, 901502, Biolegend) and Myc (1:100, sc-40, Santa Cruz), Ezrin (1:100, sc-58758, Santa Cruz), S glycoprotein (1:100, GTX632604-S, GeneTex), Nucleocapsid protein (1:100, CR3009^38^) were used. Secondary antibodies donkey anti-mouse IgG Alexa Fluor 488 (1:250, A21202, Thermo Fisher Scientific), donkey anti-mouse IgG Alexa Fluor 555 (1:250, A31570, Thermo Fisher Scientific), donkey anti-rabbit IgG Alexa Fluor 488 (1:250, A21206, Thermo Fisher Scientific), donkey anti-rabbit IgG Alexa Fluor 555 (1:250, A31572, Thermo Fisher Scientific), and rabbit anti-human FITC (1:250, F0202, Dako), as well as phalloidin-iFluor 647 (1:500, ab176759, Abcam), were applied in blocking solution to samples for 1 h at RT. Images were acquired using a Leica SP8 confocal and analysed using ImageJ Fiji.

### Immunoelectron microscopy (iEM)

HeLa cells cultured on glass coverslips were fixed in 4% PFA in PBS for 20 min at RT before blocking with 1% BSA in PBS at RT. Cryostat sections of HAE cells were immunogold labelled following a modified version of the previously described methods^39^. Sections were permeabilised with 0.05% triton in PBS for 30 min at RT followed by incubating in blocking solution consisting of 1% BSA and 0.1% acetylated BSA in PBS for 30 min at RT. For cryostat sections and HeLa cells, antibodies against S glycoprotein (1:100, GTX632604-S, GeneTex), Nucleocapsid protein (1:100, CR3009^38^), and anti-HA (1:100, 901502, Biolegend) were applied in blocking solution for 2 h at RT before incubating with 5-nm protein-A-gold in blocking solution for 1 h at RT. The samples were fixed in 2.5% glutaraldehyde in 0.05 M sodium cacodylate buffer at pH 7.4 for 1 h at RT and prepared for conventional TEM as described above but excluding propylene oxide in the sample dehydration steps.

### Statistics and reproducibility

Both TEM and IF experiments of human airway cells involved generating single samples. IF and iEM labelling of these samples was repeated using three or more sections to determine that the staining was reproducible. IF, EM, and iEM experiments of transfected HeLa cells were repeated at least three times. Statistical significance was tested using paired, two-tailed Student’s *t*-tests.

### Reporting Summary

Further information on research design is available in the Nature Research Reporting Summary linked to this article.

## Supplementary information

Supplementary Information

Description of Additional Supplementary Files

Supplementary Movie 1

Supplementary Movie 2

Supplementary Movie 3

Supplementary Movie 4

Supplementary Movie 5

Supplementary Movie 6

Supplementary Movie 7

Reporting Summary

## Data Availability

The data that support this study are available from the corresponding author upon reasonable request. Electron tomography data generated in this study have been deposited in the Electron Microscopy Data Bank, www.emdataresource.org under accession codes; EMD-14366 showing a SARS-CoV-2 virion fused to the plasma membrane of a ciliated airway cell; EMD-14359, EMD-14361, and EMD-14363 demonstrating SARS-CoV-2 virions that have a budding-like profile within a viral-containing compartments; EMD-14364 and EMD-14365 indicating the presence of SARS-CoV-2 S glycoprotein on the membrane of a viral-containing compartment; EMD-14367 showing SARS-CoV-2 within a viral-containing compartment of an infected human airway epithelial cell. Source data are provided with this paper.

## Source data

Source Data
