# Supplementary material for: Ultrastructural insight into SARS-CoV-2 entry and budding in human airway epithelium
Source: Nat Commun. 2022 Mar 25;13:1609. doi: 10.1038/s41467-022-29255-y (PMC8956608; doi:10.1038/s41467-022-29255-y)
Supplement: Supplementary file 1 — Supplementary Information [file 41467_2022_29255_MOESM1_ESM.pdf]

Ultrastructural insight into SARS-CoV-2 entry and budding in human airway epithelium

Andreia L Pinto[1], Ranjit K Rai[1], Jonathan C Brown[2], Paul Griffin[1], James R Edgar[3], Anand Shah[1,4], Aran Singanayagam[2,5], Claire Hogg[1,6], Wendy S Barclay[2], Clare E Futter[7], Thomas Burgoyne[1,7*]

[1] Royal Brompton Hospital, Guy's and St Thomas' NHS Foundation Trust, London SW3 6NP, UK

[2] Department of Infectious Disease, Imperial College London, London W2 1PG, UK

[3] Department of Pathology, University of Cambridge, Cambridge CB2 1QP

[4] MRC Centre of Global Infectious Disease Analysis, Department of Infectious Disease Epidemiology, School of Public Health, Imperial College London, UK

[5] Centre for Molecular Bacteriology and Infection, Imperial College London, London SW7 2DD, UK

[6] Academic Health Sciences Centre, Imperial College, London, London SW3 6LY, UK

[7] UCL Institute of Ophthalmology, University College London, London EC1V 9EL, UK

*Corresponding author: Tel: +44 (0)20 7608 4020, E-mail: t.burgoyne@ucl.ac.uk

Supplementary figures

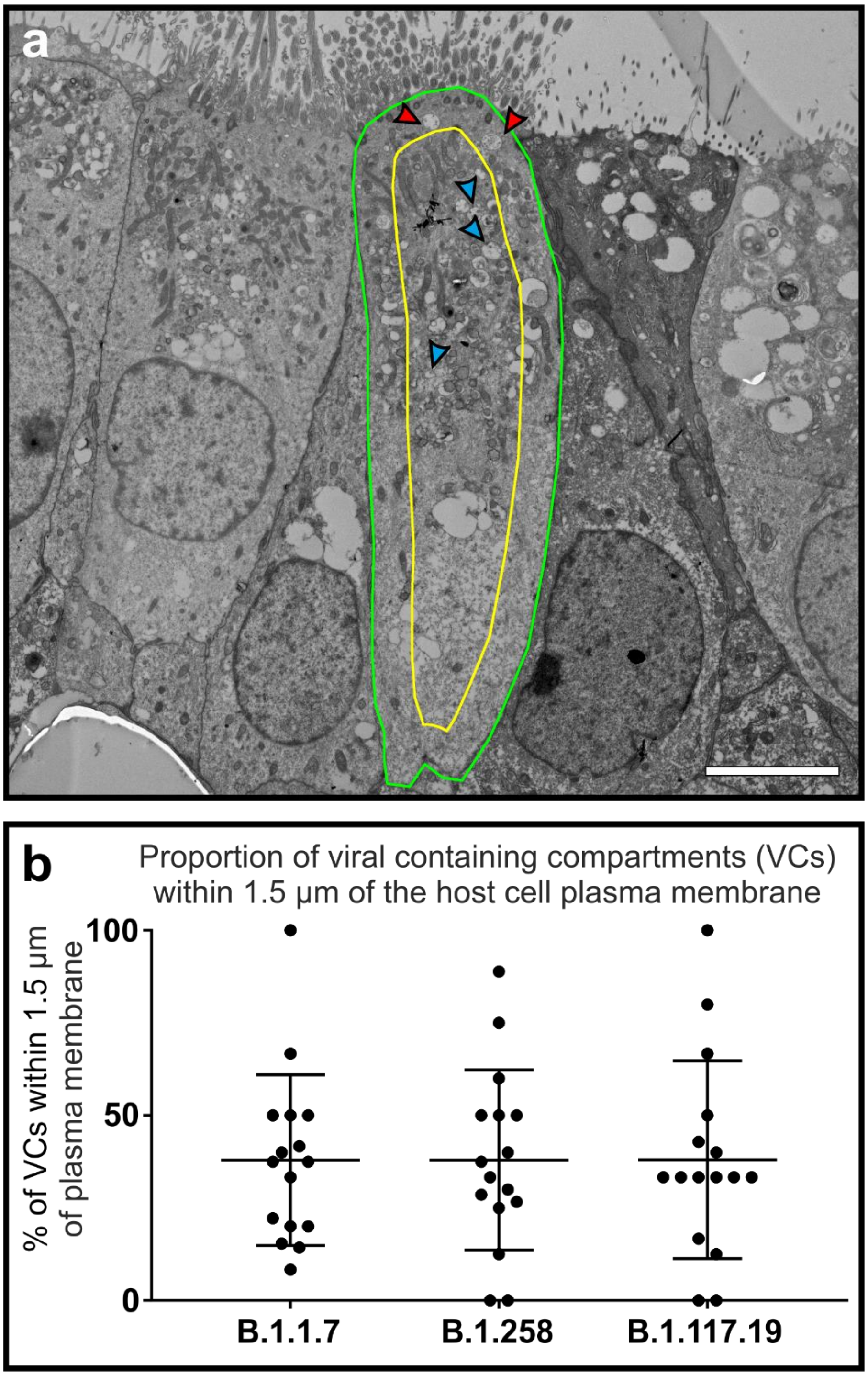

Supplementary figure 1. Quantitation of viral containing compartments (VCs) within 1.5 µm of the plasma membrane of SARS-CoV-2 infected ciliated cells. (**a**) Contours at the cell plasma membrane (green line) and 1.5 µm away from the plasma membrane (yellow line). The number of VCs between the two contours that are within 1.5 µm of host cell plasma were counted (red arrowheads) and compared to the total number of VCs within the host cell including those more than 1.5 µm away from the plasma membrane (blue arrowheads). (**b**) When comparing the percentage of VCs within 1.5 µm of host cell plasma membrane (N=16 cell), no statistical significance was determined between variants when using paired, two-tailed Student’s t-tests. Scalebar (**a**) 5 µm.

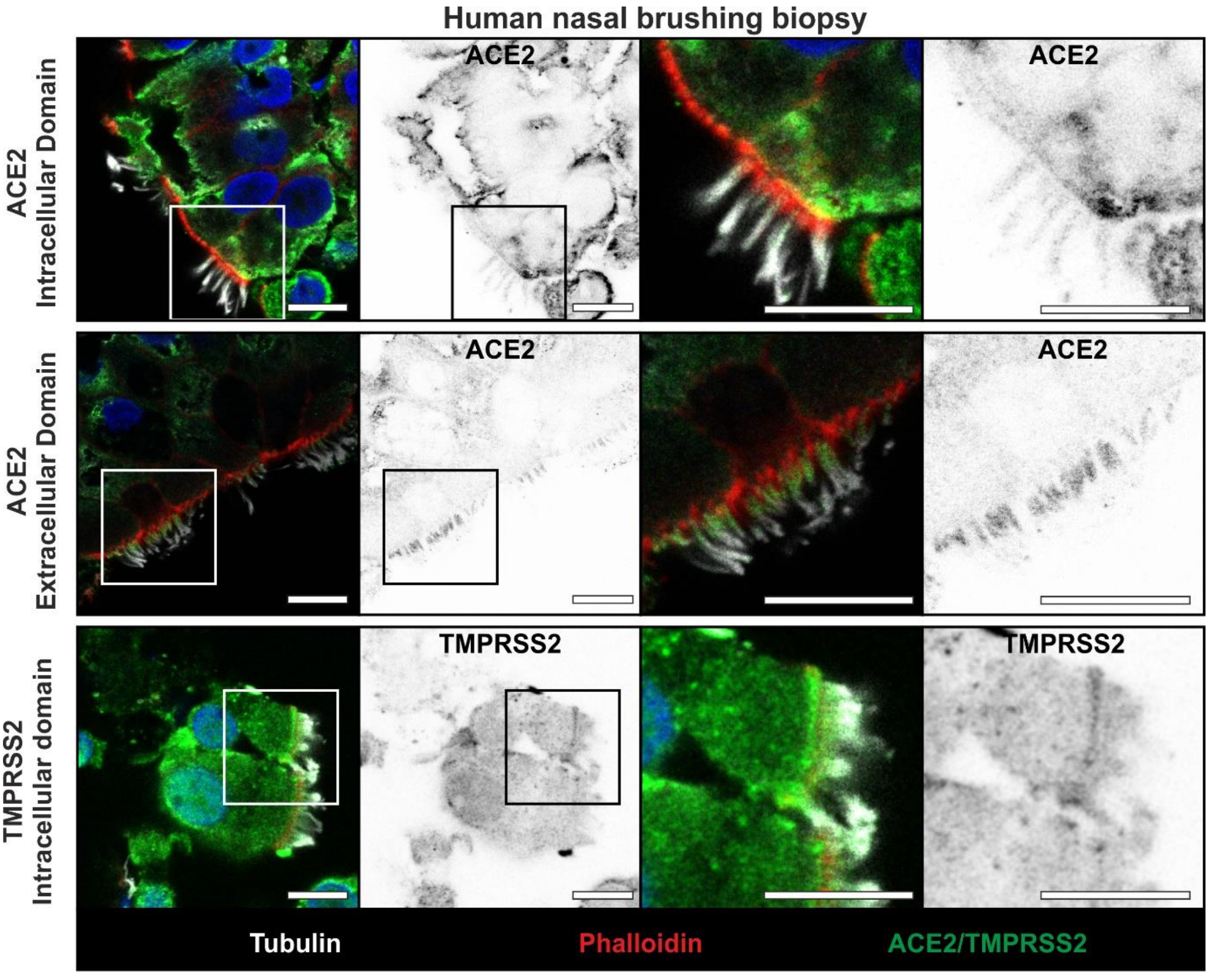

Supplementary figure 2. ACE2 and TMPRSS2 labelling of human nasal biopsy shows the same labelling pattern as cultured HAE. Scalebar 10 µm

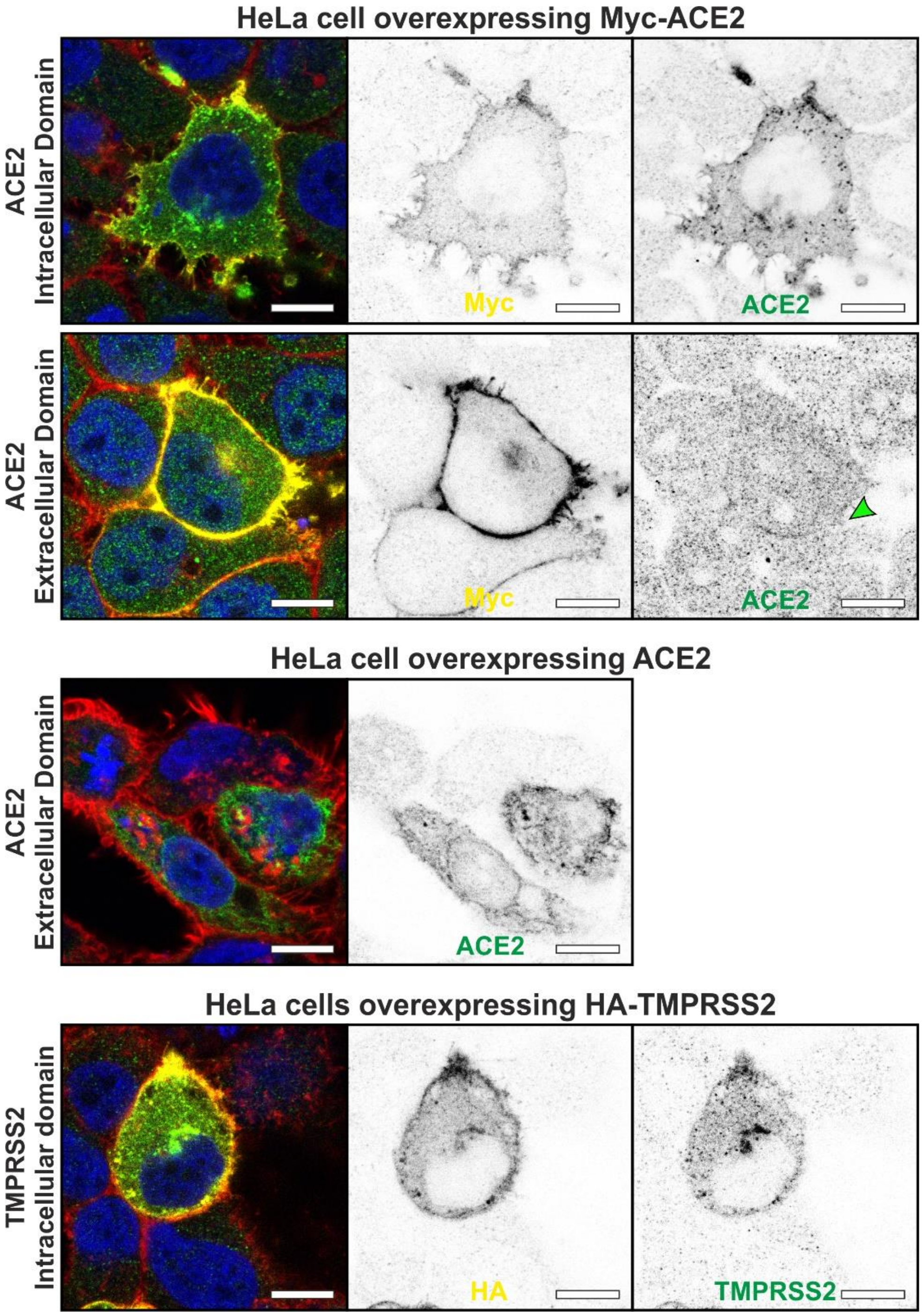

Supplementary figure 3. Validation of ACE2 and TMPRSS2 antibodies in HeLa cells expressing constructs. Signal from antibody staining overlapped with the labelling of Myc or HA in HeLa cell over expressing Myc-ACE2 or HA-TMPRSS2. For Myc-ACE2, the Myc is an N-terminal tag which is the region the extracellular domain antibody is raised against, therefore, better antibody labelling is found in HeLa cells expressing untagged ACE2. Phalloidin staining is shown in red. Scalebar 10 µm

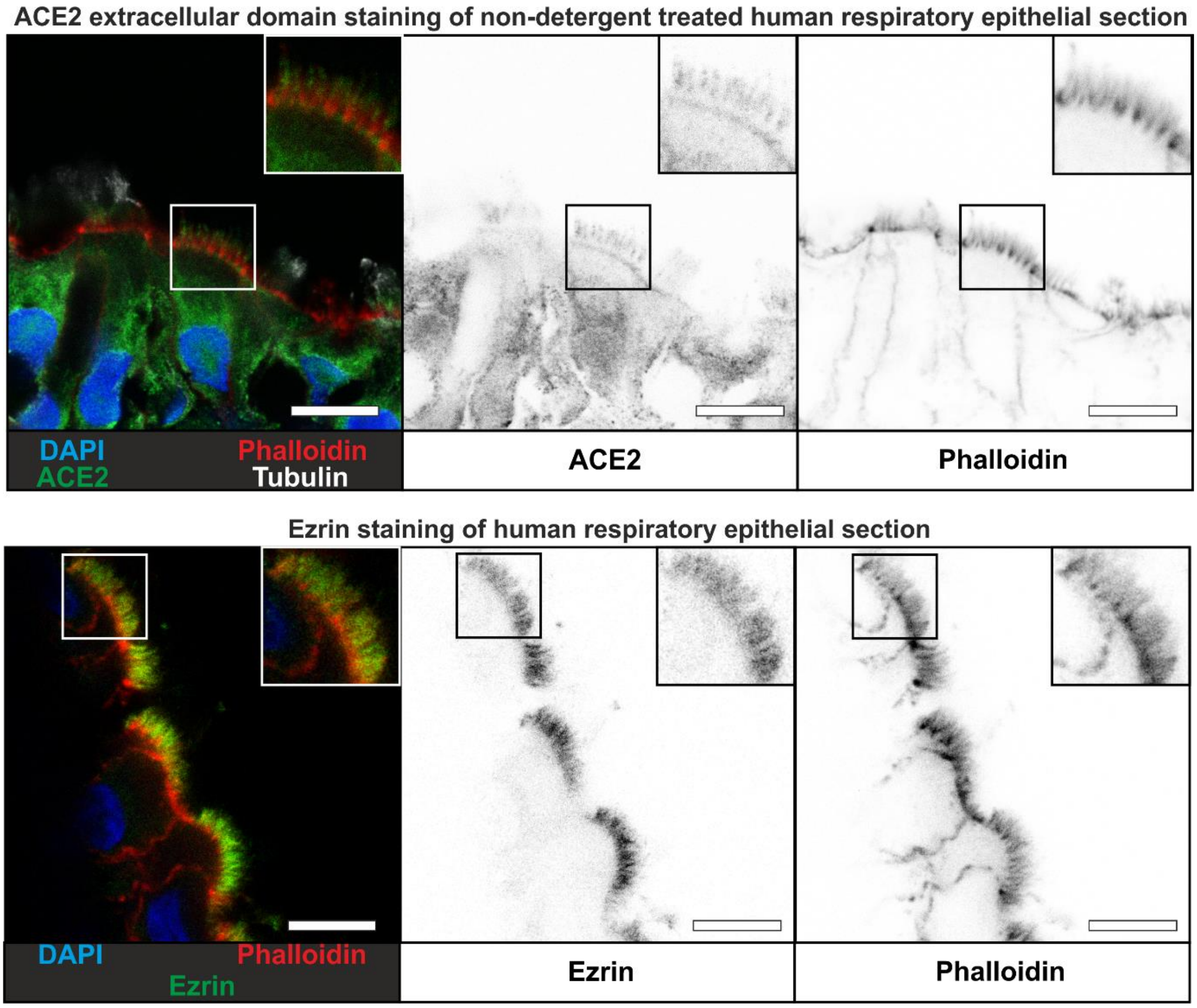

Supplementary figure 4. ACE2 antibody labelling against the extracellular domain of HAE that was not detergent-treated showed localisation to microvilli and plasma membrane. (A) The lack of detergent results in poor tubulin staining compared to permeabilised samples (see figure 2). The boxed region is an area of a ciliated cell in a slice where the cilia are absent and the ACE2 staining surrounds the actin (phalloidin stained) enriched microvilli. (B) Ezrin localises to microvilli and the staining appears to surround the actin (phalloidin stained) in HAE cells and shows some similarity to ACE2 staining. Boxed regions are shown at higher magnification in the top righthand panels. Scalebars 10 µm

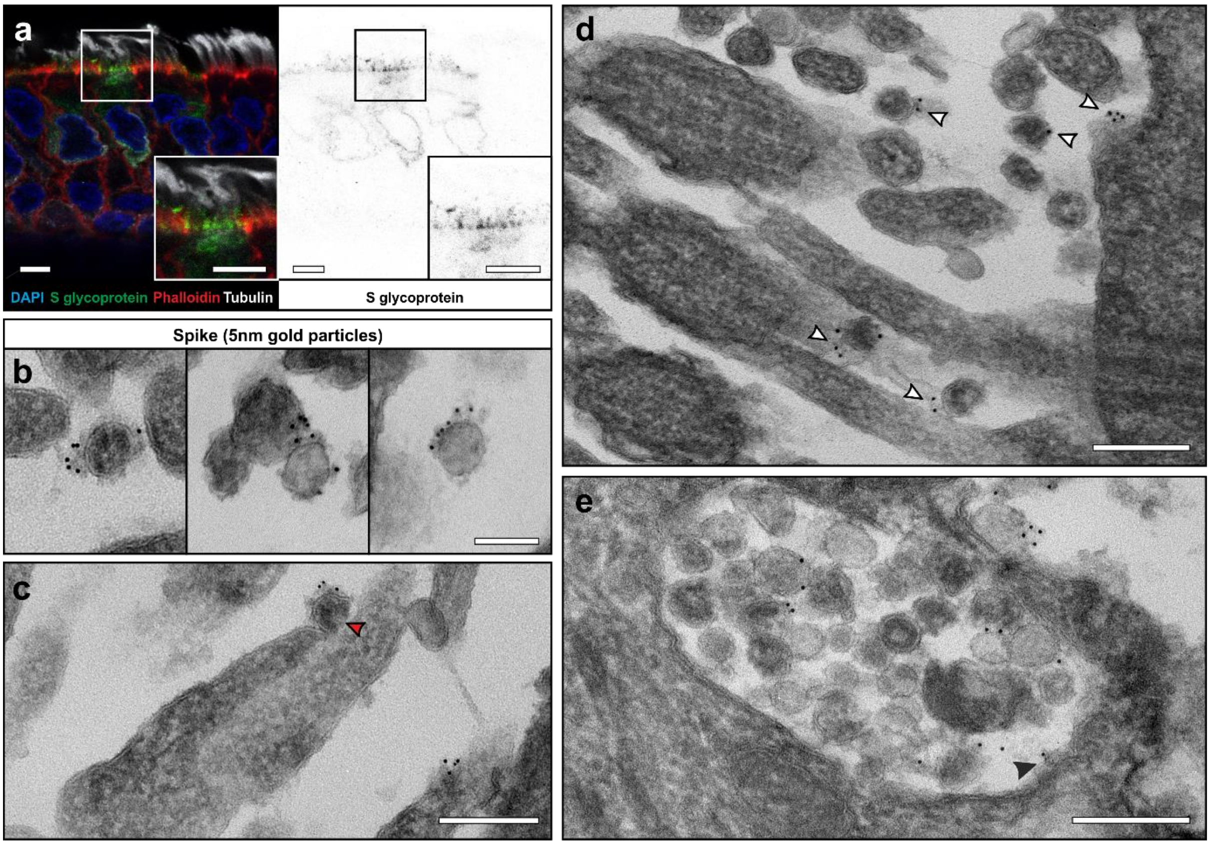

Supplementary figure 5. S glycoprotein is enriched on the plasma membrane apart from cilia and within viral containing compartments of SARS-CoV-2 (B.1.258) infected HAE cells. (**a**) Immunofluorescence antibody labelling against S glycoprotein (in green) shows that it is enriched at the cell surface were there are actin enriched microvilli (phalloidin staining in red) but does not colocalise with cilia (stained with anti-tubulin antibody and shown in white). (**b** - **e**) Immuno electron microscopy gold labelling of S glycoprotein in an HAE sample infected with SARS-CoV-2. (**b**) Virions with gold labelled S glycoprotein at the surface. (**c**) A microvillus that has a viral fusion like profile (red arrowhead) that labels for S glycoprotein. (**d**) Microvilli and other regions of the cell surface (white arrowheads) showing presence of gold labelled S glycoprotein. (**e**) A viral containing compartment (VC) with gold localised to the organelle membrane (black arrowhead). Scalebars (**a**) 5 µm (**b**) 100nm (**c** - **e**) 200 nm.

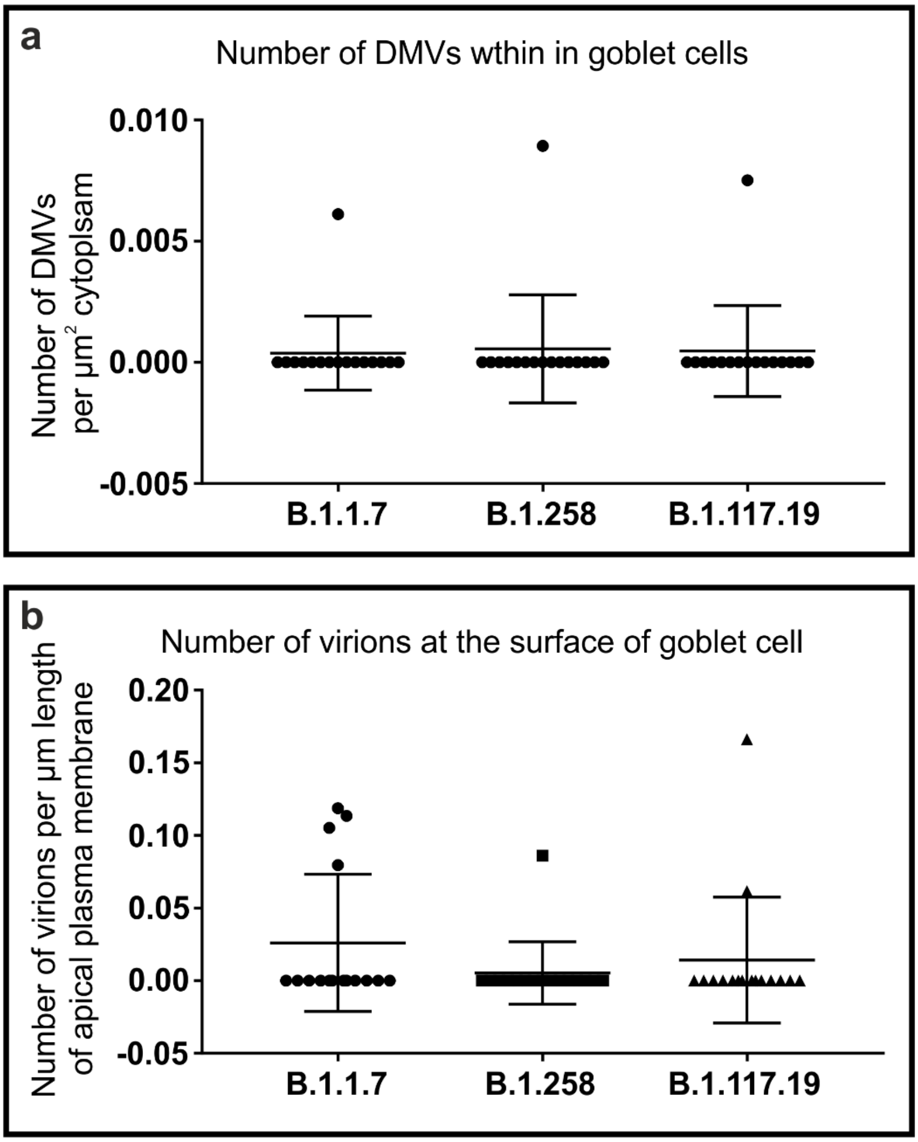

Supplementary figure 6. Further evidence that goblet cells are not susceptible to infection by SARS-CoV-2. (**a**) A small number of double membrane vesicles (DMVs) were seen within goblet cells. (**b**) Few virions were detected at the surface of goblet cells. (**a** & **b**) N=16 and no statistical significance was determine using paired, two-tailed Student's t-tests.

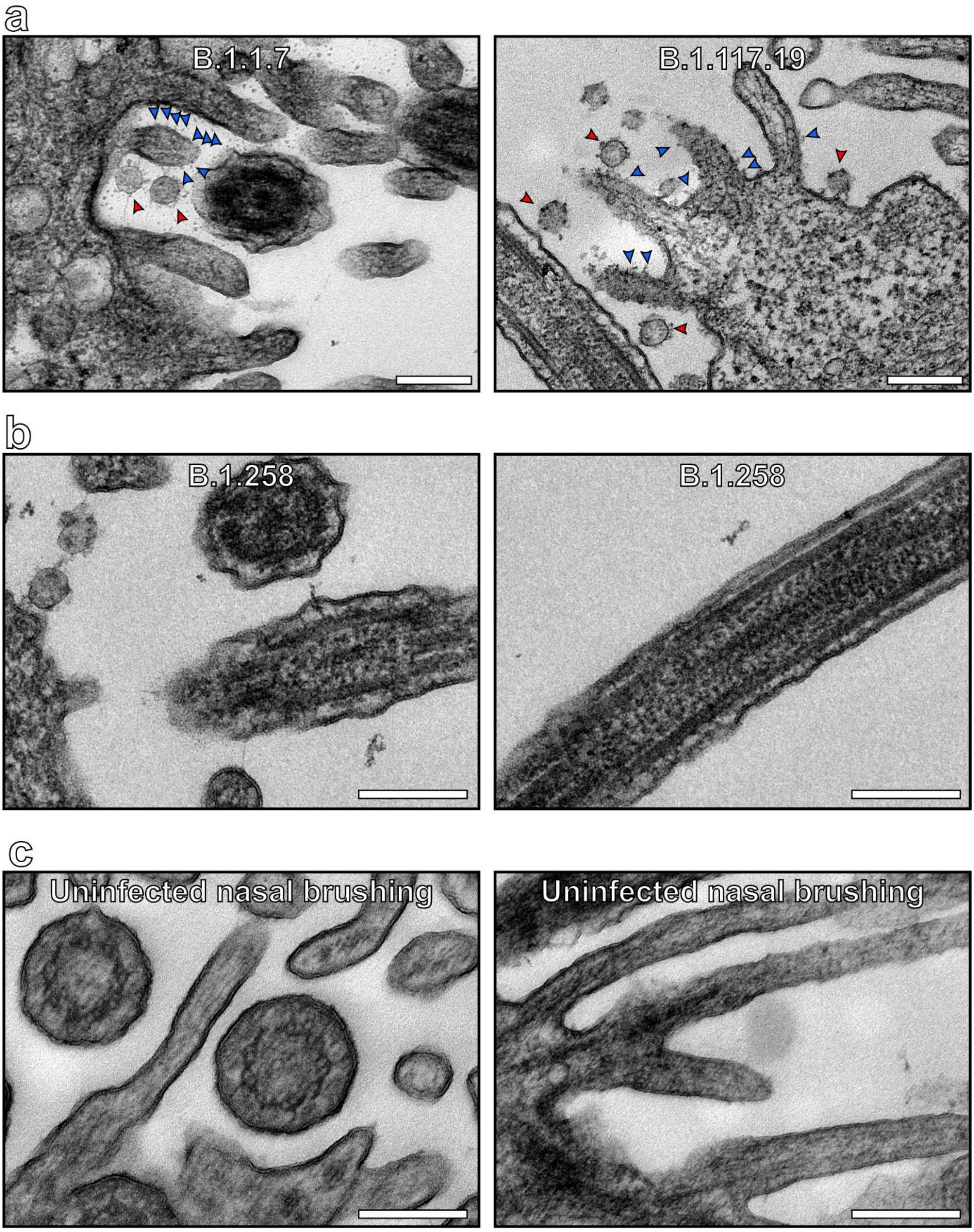

Supplementary figure 7. Infected ciliated HAE cells have protrusions on the plasma membrane that exclude cilia. (**a**) Protrusions seen on the plasma membrane of microvilli (blue arrowheads) of infected (virions shown by red arrowheads) ciliated cells. (**b**) Ciliary membranes of an infected cell are smooth. (**c**) Microvilli and other regions of the plasma membrane are smooth in an uninfected nasal brushing sample. Scalebars 200 nm

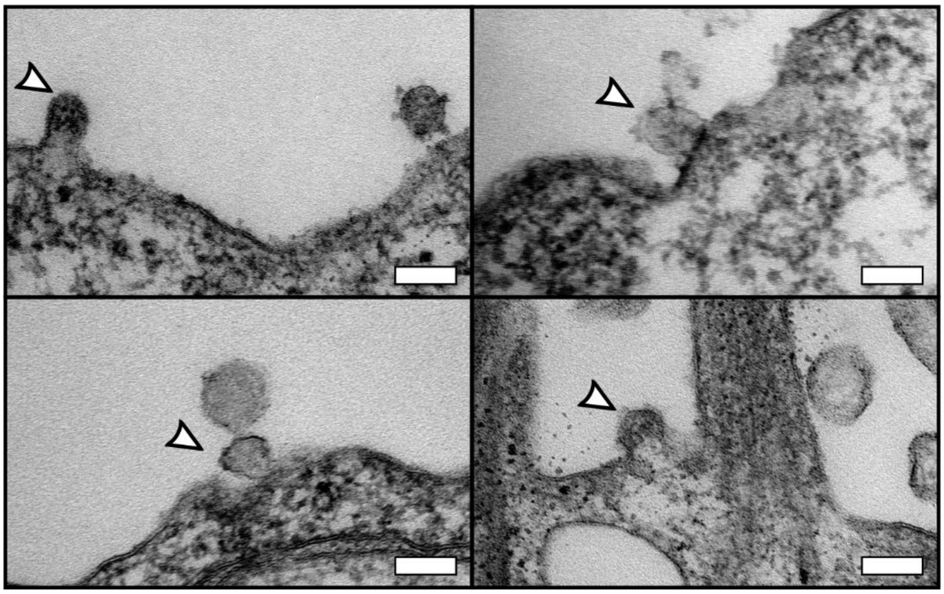

Supplementary figure 8. Further examples of SARS-CoV-2 virions fusing at the plasma membrane of host HAE cells (indicated by white arrowheads). Scale bar 100nm.

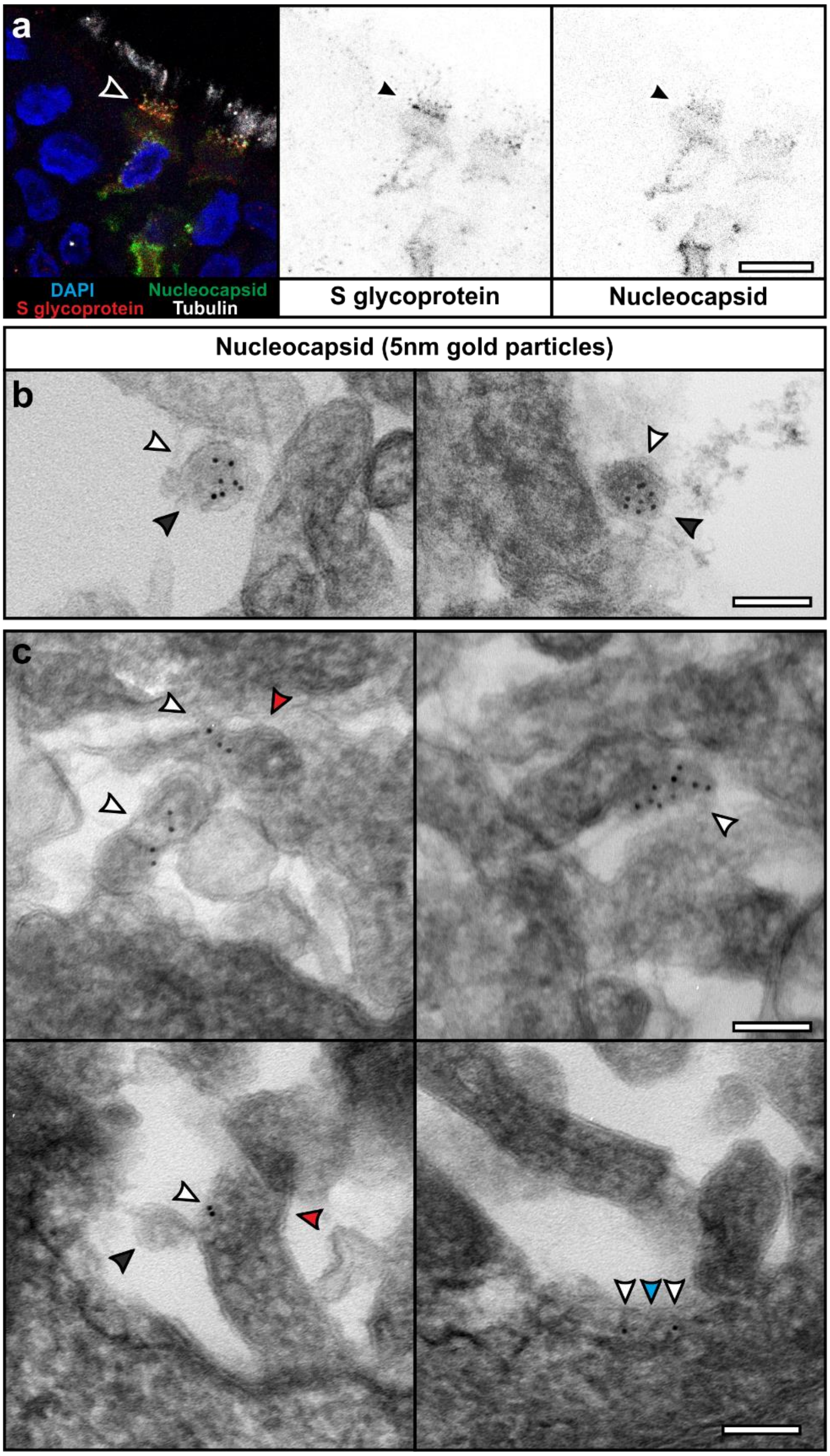

Supplementary figure 9. Nucleocapsid protein is found within SARS-CoV-2 (B.1.258) virions and infected cells including at the plasma membrane. (**a**) Immunofluorescence antibody labelling of S-glycoprotein and nucleocapsid protein shows that the two colocalise and there is staining at the cell plasma membrane as indicated by the black arrowheads. (**b** - **c**) Immuno electron microscopy antibody labelling of nucleocapsid protein. (**b**) Nucleocapsid immuno gold labelling localised within virions. (**c**) Immuno gold labelling shows the presence of nucleocapsid within microvilli (red arrowheads) including close to a structure that resembles a fusing virion (black arrowhead) and at the cell surface (blue arrowhead) in infected HAE cells. Scalebar (**a**) 10 µm (**b** & **c**) 100 nm.

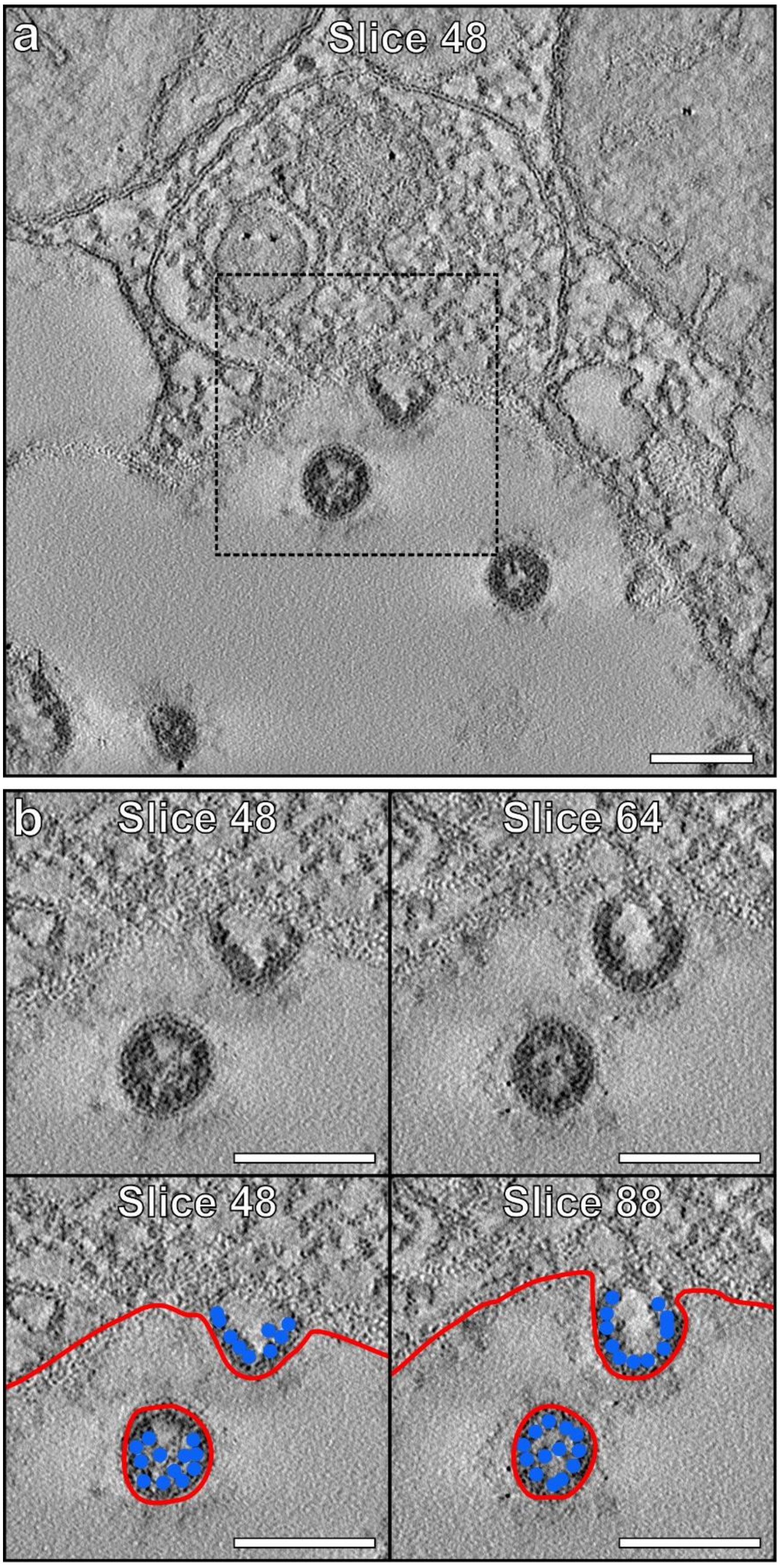

Supplementary figure 10. Nucleocapsid protein appears to concentrate at the membrane of virions with a budding profile within VCs. (**a** – **b**) Slices from a tomogram that shows a virion with a budding profile as well as discrete virions within VCs. The boxed region in (**a**) is shown at higher magnification in (**b**). (**b**) Nucleocapsid protein false coloured in blue appears to be concentrated at the membrane of virions with a budding profile that are connected to the VC membrane (coloured in red). Within the discrete virion the nucleocapsid protein appears to be more dispersed. Scalebars 100 nm.

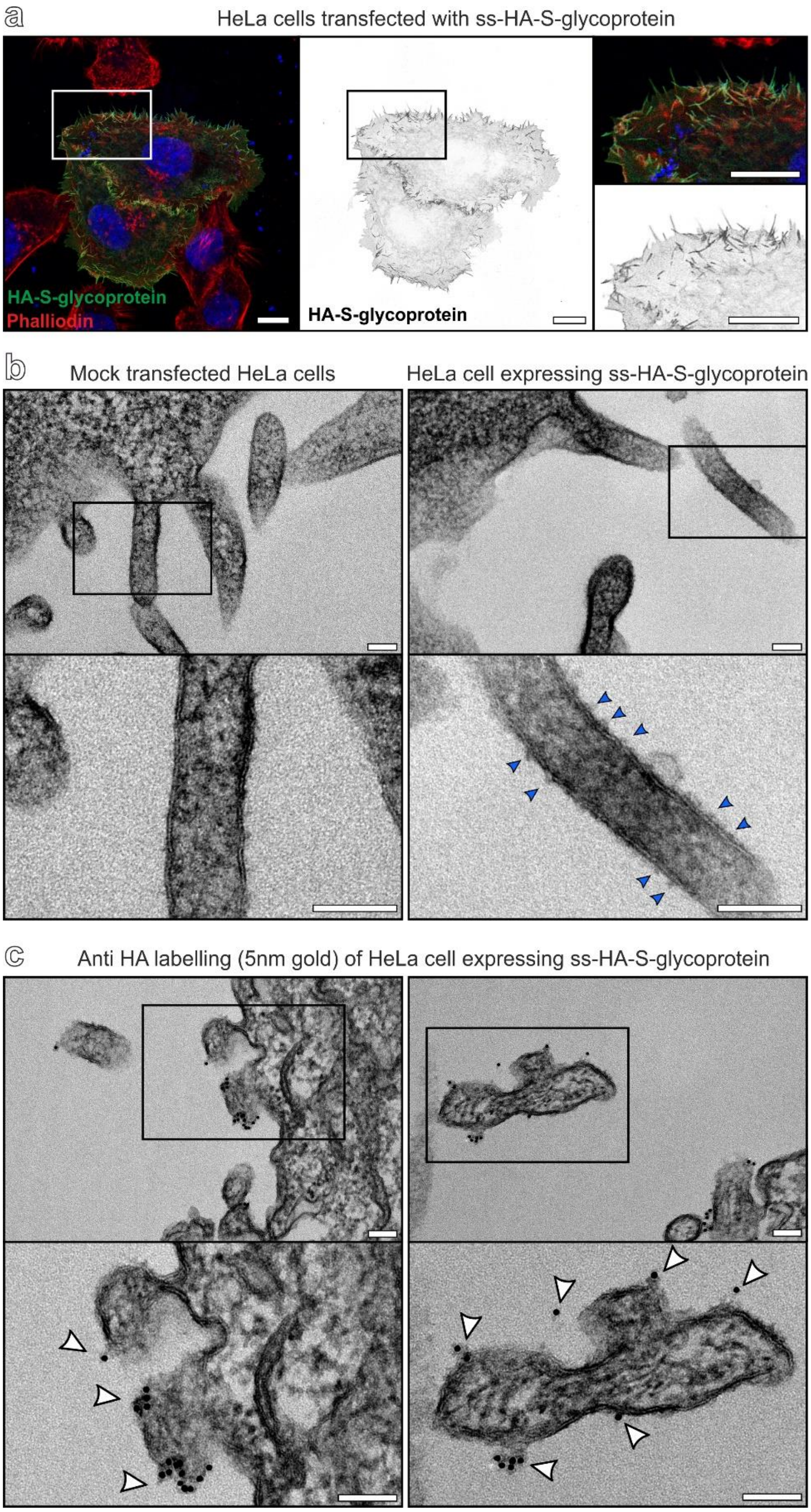

Supplementary figure 11. Overexpression of ss-HA-S-glycoprotein in HeLa cells leads to it being localised to the plasma membrane including to microvilli and results in protrusions as seen by EM. (**a**) IF images showing HA-S-glycoprotein localised to the plasma membrane in over expressing HeLa cells. (**b**) HeLa cells over expressing ss-HA-S-glycoprotein have protrusions on the microvilli when compared to mock transfected cells. (**c**) Anti HA gold particle labelling localises to protrusions at the cell surface by immuno electron microscopy (white arrowheads). Scalebars (**a**) 10 µm and (**b** & **c**) 100 nm.
