# Supplementary material for: Ultrastructural insight into SARS-CoV-2 entry and budding in human airway epithelium
Source: Nat Commun. 2022 Mar 25;13:1609. doi: 10.1038/s41467-022-29255-y (PMC8956608; doi:10.1038/s41467-022-29255-y)
Supplement: Supplementary file 2 — Description of Additional Supplementary Files [file 41467_2022_29255_MOESM2_ESM.pdf]

Description of Additional Supplementary Files

File Name: Supplementary Movie 1
Description: Tomogram of a SARS-CoV-2 virion fused to the plasma membrane of a ciliated airway cell.

File Name: Supplementary Movie 2
Description: Tomogram of virions that appear to be budding into a viral containing compartment.

File Name: Supplementary Movie 3
Description: Further example of virions that appear to be budding into a viral containing compartment.

File Name: Supplementary Movie 4
Description: Higher magnification example of virions that appear to be budding into a viral containing compartment.

File Name: Supplementary Movie 5
Description: Tomogram showing S glycoprotein protrusion on viral containing compartment limiting membrane.

File Name: Supplementary Movie 6
Description: Further example of S glycoprotein protrusion on viral containing compartment limiting membrane.

File Name: Supplementary Movie 7
Description: Tomogram of virions within a viral containing compartment.
