# Supplementary material for: Ultrastructural insight into SARS-CoV-2 entry and budding in human airway epithelium
Source: Nat Commun. 2022 Mar 25;13:1609. doi: 10.1038/s41467-022-29255-y (PMC8956608; doi:10.1038/s41467-022-29255-y)
Supplement: Supplementary file 10 — Reporting Summary [file 41467_2022_29255_MOESM10_ESM.pdf]

nature research

Corresponding author(s): Thomas Burgoyne

Last updated by author(s): Jan 17, 2022

Reporting Summary

Nature Research wishes to improve the reproducibility of the work that we publish. This form provides structure for consistency and transparency in reporting. For further information on Nature Research policies, see our Editorial Policies and the Editorial Policy Checklist.

Statistics

For all statistical analyses, confirm that the following items are present in the figure legend, table legend, main text, or Methods section.

n/a	Confirmed	
☐	☒	The exact sample size (*n*) for each experimental group/condition, given as a discrete number and unit of measurement
☐	☒	A statement on whether measurements were taken from distinct samples or whether the same sample was measured repeatedly
☐	☒	The statistical test(s) used AND whether they are one- or two-sided<br>*Only common tests should be described solely by name; describe more complex techniques in the Methods section.*
☒	☐	A description of all covariates tested
☒	☐	A description of any assumptions or corrections, such as tests of normality and adjustment for multiple comparisons
☐	☒	A full description of the statistical parameters including central tendency (e.g. means) or other basic estimates (e.g. regression coefficient) AND variation (e.g. standard deviation) or associated estimates of uncertainty (e.g. confidence intervals)
☐	☒	For null hypothesis testing, the test statistic (e.g. *F*, *t*, *r*) with confidence intervals, effect sizes, degrees of freedom and *P* value noted<br>*Give P values as exact values whenever suitable.*
☒	☐	For Bayesian analysis, information on the choice of priors and Markov chain Monte Carlo settings
☒	☐	For hierarchical and complex designs, identification of the appropriate level for tests and full reporting of outcomes
☒	☐	Estimates of effect sizes (e.g. Cohen's *d*, Pearson's *r*), indicating how they were calculated

Our web collection on statistics for biologists contains articles on many of the points above.

Software and code

Policy information about availability of computer code

Data collection	Transmission electron microscopy images were acquired using AMT Capture Engine (version 7.00) and Gatan Digital Micrograph (version 2.32.866.0), tomography tilt series using SerialEM (version 3.7.0 developed at University of Colorado, Boulder) and confocal images collected on a Leica SP8 with Leica Application Suite X (version 3.5.7.23225).
Data analysis	Tomography data was prepared and analysed in IMOD (version 4.9.12 developed at University of Colorado, Boulder) and subtomographic reconstruction generated in the IMOD PEET package (version 1.14.0 developed at University of Colorado, Boulder) and viewed in Chimera (version 1.14 developed at UCSF). Image data (electron microscopy and confocal) was analyzed and measurements made using ImageJ Fiji (version 1.53f51). Measurements were assessed in Microsoft Excel 365 and Graphpad Prism (version 7).

For manuscripts utilizing custom algorithms or software that are central to the research but not yet described in published literature, software must be made available to editors and reviewers. We strongly encourage code deposition in a community repository (e.g. GitHub). See the Nature Research guidelines for submitting code & software for further information.

Data

Policy information about availability of data

All manuscripts must include a data availability statement. This statement should provide the following information, where applicable:

- Accession codes, unique identifiers, or web links for publicly available datasets
- A list of figures that have associated raw data
- A description of any restrictions on data availability

Source data for Figure 1 - 4 and Supplementary Figures 1 and 6 are provided with the paper. Electron tomography data generated in this study have been deposited in the Electron Microscopy Data Bank, www.emdataresource.org under the following accession codes; EMD-14366 showing a SARS-CoV-2 virion fused to the plasma membrane of a ciliated airway cell; EMD-14359, EMD-14361 and EMD-14363 demonstrating SARS-CoV-2 virions that have a budding like profile within a viral

containing compartments; EMD-14364 and EMD-14365 indicating the presence of SARS-CoV-2 S glycoprotein on the membrane of a viral containing compartment; EMD-14367 showing SARS-CoV-2 within a viral containing compartment of an infected human airway epithelial cell.

Field-specific reporting

Please select the one below that is the best fit for your research. If you are not sure, read the appropriate sections before making your selection.

☒ Life sciences ☐ Behavioural & social sciences ☐ Ecological, evolutionary & environmental sciences

For a reference copy of the document with all sections, see nature.com/documents/nr-reporting-summary-flat.pdf

Life sciences study design

All studies must disclose on these points even when the disclosure is negative.

Sample size	No statistical method was used to predetermine sample size as this study compared features of infection found between different SARS-CoV-2 variants rather than comparing human or animal subjects.<br><br>We selected sample sizes based on published studies of organelles by electron microscopy and our previous experience examining endosomes that have a size and morphology similar to viral containing compartments (see Höglinger et al, 2019, Burgoyne et al, 2013 and Tomas et al, 2015). There was a clear difference in the number of viral containing compartments between goblet and ciliated cells as well as the number of virions attached to microvilli compared to cilia. The number of cells analyzed in these experiments gave high significance indicating the sample sizes were more than sufficient. For all other experiments that did not involve quantification, they were repeated at least 3 times which is commonly accepted best practice to determine that the features seen, and labelling (immunofluorescence and immunoEM) were consistent and reproducible.
Data exclusions	No data was excluded from analysis.
Replication	Both electron microscopy (EM) and immunofluorescence (IF) experiments of human airway cells were from single samples of cells infected with three different SARS-CoV-2 variants. Multiple EM sections from all samples were studied and similar results were found for the three variants. IF and immunoEM (iEM) experiments were repeated on at least 3 sections and all attempts confirmed the same findings. IF, EM and iEM experiments of transfected HeLa cell were repeat at least three times and the same findings were found for all repeats.
Randomization	By EM, random cells were selected and imaged for quantification and analysis. When comparing goblet against ciliated cells and SARS-CoV-2 attachment to cilia or microvilli, images could not be randomised as these cell types and organelles are morphologically identifiable within EM images. When viewing sections prepared for IF and iEM, entire strips of cells were examined to determine whether findings were consistent across a large number of cells.
Blinding	No blinding was performed during data collection as the different SARS-CoV-2 variants gave infected samples that appeared indistinguishable from each other when viewing on a microscope. Each SARS-CoV-2 infected sample was given a code that was unknown to the member of the lab doing the quantitative analysis from the electron microscopy images. The identity of the SARS-CoV-2 variant was only provided after the analysis.

Reporting for specific materials, systems and methods

We require information from authors about some types of materials, experimental systems and methods used in many studies. Here, indicate whether each material, system or method listed is relevant to your study. If you are not sure if a list item applies to your research, read the appropriate section before selecting a response.

Materials & experimental systems

n/a	Involved in the study	
☐	☒	Antibodies
☐	☒	Eukaryotic cell lines
☒	☐	Palaeontology and archaeology
☒	☐	Animals and other organisms
☐	☒	Human research participants
☒	☐	Clinical data
☒	☐	Dual use research of concern

Methods

n/a	Involved in the study	
☒	☐	ChIP-seq
☒	☐	Flow cytometry
☒	☐	MRI-based neuroimaging

Antibodies

Antibodies used	The following antibodies were used in this study against:<br>α Tubulin (mouse monoclonal) Santa Cruz – sc-32293 (DM1A) (1:250 for IF)<br>α Tubulin (rabbit monoclonal) Abcam – ab52866 (1:250 for IF)<br>ACE2 (rabbit polyclonal) Abcam - ab15348 (1:100 for IF)

ACE2 (rabbit polyclonal) Sigma-Aldrich - HPA000288 (1:100 for IF)
TMPRSS2 (rabbit polyclonal) Novus Biologicals - NBP2-38263 (1:100 for IF)
HA Biolegend (mouse monoclonal) - 901502 (16B12) (1:100 for IF)
Myc (mouse monoclonal) Santa Cruz - sc-40 (9E10) (1:100 for IF)
Ezrin (mouse monoclonal) Santa Cruz - sc-58758 (3C12) (1:100 for IF)
S glycoprotein (mouse monoclonal) GeneTex - GTX632604-S (1:100 for IF and iEM)
Nucleocapsid protein (human monoclonal) provided by Annabel Borg and Svend Kjaer at the Crick Institute (Zeng et al., 2021) - CR3009 (1:100 for IF and iEM)
Rabbit anti-human IgG FITC secondary antibody Dako - F0202 (1:250 for IF)
Donkey anti-mouse IgG Alexa Fluor 488 secondary antibody Thermo Fisher Scientific – A21202 (1:250 for IF)
Donkey anti-mouse IgG Alexa Fluor 555 secondary antibody Thermo Fisher Scientific – A31570 (1:250 for IF)
Donkey anti-rabbit IgG Alexa Fluor 488 secondary antibody Thermo Fisher Scientific – A21206 (1:250 for IF)
Donkey anti-rabbit IgG Alexa Fluor 555 secondary antibody Thermo Fisher Scientific – A31572 (1:250 for IF)

Validation

α Tubulin (mouse monoclonal) Santa Cruz – sc-32293
Validated by IF (1:250)
Link to manufacturer’s website https://www.scbt.com/p/alpha-tubulin-antibody-dm1a
Referenced in 530 publications

α Tubulin (rabbit monoclonal) Abcam – ab52866
Validated by IF (1:250)
Link to manufacturer’s website https://www.abcam.com/alpha-tubulin-antibody-ep1332y-microtubule-marker-ab52866.html
Referenced in 169 publications

ACE2 Abcam - ab15348
Validated by IF (1:100)
Link to manufacturer’s website https://www.abcam.com/ace2-antibody-ab15348.html
Referenced in 26 publications

ACE2 Sigma-Aldrich - HPA000288
Validated by IF (1:100)
Link to manufacturer’s website https://www.sigmaaldrich.com/GB/en/product/sigma/hpa000288
Manufacturer states: All Prestige Antibodies®Powered by Atlas Antibodies is developed and validated by the Human Protein Atlas (HPA) project (www.proteinatlas.org). Each antibody is tested by immunohistochemistry against hundreds of normal and disease tissues. These images can be viewed on the Human Protein Atlas (HPA) site by clicking on the Image Gallery link. To view these protocols and other useful information about Prestige Antibodies and the HPA, visit sigma.com/prestige.
Referenced in 22 publications

TMPRSS2 (Novus Biologicals - NBP2-38263)
Validated by IF (1:100)
Link to manufacturer’s website https://www.novusbio.com/products/tmprss2-antibody_nbp2-38263
Manufacturer states: The antibody was validated by orthogonal strategies- The target protein is examined with an antibody independent strategy and compared with results from an antibody-dependent strategy. A correlation between these two strategies indicates specificity between the antibody and its target protein. Examples of antibody independent techniques may include in situ hybridization, quantitative PCR, RNA-seq or mass spectrometry.
Referenced in 1 publication

HA (Biolegend - 901502)
Validated by IF (1:100)
Link to manufacturer’s website https://www.biolegend.com/en-us/products/purified-anti-ha-11-epitope-tag-antibody-11374
Referenced in 424 publications

Myc (Santa Cruz - sc-40)
Validated by IF (1:100)
Link to manufacturer’s website https://www.scbt.com/p/c-myc-antibody-9e10
Referenced in 8686 publications

Ezrin (Santa Cruz - sc-58758)
Validated by IF (1:100)
Link to manufacturer’s website https://www.scbt.com/p/ezrin-antibody-3c12
Referenced in 52 publications

S glycoprotein (GeneTex - GTX632604-S)
Validated by IF (1:100)
Link to manufacturer’s website https://www.genetex.com/Product/Detail/SARS-CoV-SARS-CoV-2-COVID-19-spike-antibody-1A9/GTX632604
Referenced in approximately 130 publications

Nucleocapsid protein (CR3009)

	Validated by IF (1:100)<br>This antibody was developed and tested as described in the following paper:<br>Zeng, J., et al 2021. Identifying SARS-CoV-2 antiviral compounds by screening for small molecule inhibitors of nsp13 helicase. Biochem J. 478:2405–2423. doi:10.1042/BCJ20210201.<br><br>Donkey anti-mouse IgG Alexa Fluor 488 secondary antibody (Thermo Fisher Scientific – A21202)<br>Validated by IF (1:250)<br>Link to manufacturer's website https://www.thermofisher.com/antibody/product/Donkey-anti-Mouse-IgG-H-L-Highly-Cross-Adsorbed-Secondary-Antibody-Polyclonal/A-21202<br><br>Donkey anti-mouse IgG Alexa Fluor 555 secondary antibody (Thermo Fisher Scientific – A31570)<br>Validated by IF (1:250)<br>Link to manufacturer's website https://www.thermofisher.com/antibody/product/Donkey-anti-Mouse-IgG-H-L-Highly-Cross-Adsorbed-Secondary-Antibody-Polyclonal/A-31570<br><br>Donkey anti-rabbit IgG Alexa Fluor 488 secondary antibody (Thermo Fisher Scientific – A21206)<br>Validated by IF (1:250)<br>Link to manufacturer's website https://www.thermofisher.com/antibody/product/Donkey-anti-Rabbit-IgG-H-L-Highly-Cross-Adsorbed-Secondary-Antibody-Polyclonal/A-21206<br><br>Donkey anti-rabbit IgG Alexa Fluor 555 secondary antibody (Thermo Fisher Scientific – A31572)<br>Validated by IF (1:250)<br>Link to manufacturer's website https://www.thermofisher.com/antibody/product/Donkey-anti-Rabbit-IgG-H-L-Highly-Cross-Adsorbed-Secondary-Antibody-Polyclonal/A-31572

Eukaryotic cell lines

Policy information about cell lines

Cell line source(s)	HeLa cells from ATCC (CCL2)
Authentication	Cell line was not authenticated
Mycoplasma contamination	Cell line had no mycoplasma contamination as tested using DAPI
Commonly misidentified lines (See ICLAC register)	No commonly misidentified cell lines were used

Human research participants

Policy information about studies involving human research participants

Population characteristics	Adults (18+) were recruited and a specific sex was not required for this study. To gain airway cells a single healthy participant without respiratory disease was required. To study an SARS-CoV-2 infected nasal brushing sample a single SARS-CoV-2 infected patient was required that had a positive PCR result for SARS-CoV-2.
Recruitment	Participants were recruited by informed consented. A single healthy control participant provided a nasal brushing biopsy sample to allow cells to be cultured for all in vitro infection experiments. As the study compares different SARS-CoV-2 variants within cells from a single donor, selection bias was not relevant for these experiments. A single SARS-CoV-2 infected subject that tested positive for the virus by PCR, provided a sample that was compared in this study to in vitro infection experiments. No selection bias was involved.
Ethics oversight	This study complies to ethical regulations for acquiring and use of human tissue under Health Research Authority (HRA) study approval (REC ref: 20/SC/0208; IRAS: 282739) and informed consent was obtained from the participants.

Note that full information on the approval of the study protocol must also be provided in the manuscript.
